# Short-chain fatty acid-producing bacterial strains attenuate experimental ulcerative colitis by promoting M2 macrophage polarization via JAK/STAT3/FOXO3 axis inactivation

**DOI:** 10.1186/s12967-024-05122-w

**Published:** 2024-04-18

**Authors:** Hailan Zhao, Youlian Zhou, Jing Xu, Yong Zhang, Hong Wang, Chong Zhao, Hongli Huang, Jing Yang, Chen Huang, Yingfei Li, Lisheng Wang, Yuqiang Nie

**Affiliations:** 1 Department of Gastroenterology and Hepatology, Guangzhou First People’s Hospital, the Second Affiliated Hospital, School of Medicine, South China University of Technology, Guangzhou, 510006 China; 2 Department of Gastroenterology, Shenzhen People’s Hospital (The Second Clinical Medical College, Jinan University; The First Affiliated Hospital, Southern University of Science and Technology), Shenzhen, 518020 China; 3 The First Affiliated Hospital, Jinan University, Guangzhou, 510630 China; 4 Department of Pathology, Guangzhou First People’s Hospital, Guangzhou, 510180 China

**Keywords:** Short-chain fatty acids, Bacterial mixture, Macrophage polarization, Colitis

## Abstract

**Background:**

Patients with inflammatory bowel disease (IBD), dysbiosis, and immunosuppression who receive fecal microbiota transplantation (FMT) from healthy donors are at an increased risk of developing bacteremia. This study investigates the efficacy of a mixture of seven short-chain fatty acid (SCFA)-producing bacterial strains (7-mix), the resulting culture supernatant mixture (mix-sup), and FMT for treating experimental ulcerative colitis (UC) and evaluates underlying mechanisms.

**Methods:**

Utilizing culturomics, we isolated and cultured SCFA-producing bacteria from the stool of healthy donors. We used a mouse model of acute UC induced by dextran sulfate sodium (DSS) to assess the effects of 7-mix, mix-sup, and FMT on intestinal inflammation and barrier function, microbial abundance and diversity, and gut macrophage polarization by flow cytometry, immunohistochemistry, 16S rRNA gene sequencing, and transwell assays.

**Results:**

The abundance of several SCFA-producing bacterial taxa decreased in patients with UC. Seven-mix and mix-sup suppressed the inflammatory response and enhanced intestinal mucosal barrier function in the mouse model of UC to an extent similar to or superior to that of FMT. Moreover, 7-mix and mix-sup increased the abundance of SCFA-producing bacteria and SCFA concentrations in colitic mice. The effects of these interventions on the inflammatory response and gut barrier function were mediated by JAK/STAT3/FOXO3 axis inactivation in macrophages by inducing M2 macrophage polarization in vivo and in vitro.

**Conclusions:**

Our approach provides new opportunities to rationally harness live gut probiotic strains and metabolites to reduce intestinal inflammation, restore gut microbial composition, and expedite the development of safe and effective treatments for IBD.

**Supplementary Information:**

The online version contains supplementary material available at 10.1186/s12967-024-05122-w.

## Introduction

Inflammatory bowel disease (IBD) presents in two clinical forms—ulcerative colitis (UC) and Crohn’s disease (CD)—and is characterized by chronic relapsing and remitting inflammation in the gastrointestinal tract. IBD is associated with gut barrier dysfunction and intestinal dysbiosis, which induce the uncontrolled activation of immune cells in genetically susceptible individuals [1, 2].

Macrophage polarization in response to stimuli occurs as a result of the signal transduction and transcriptional regulation functions of Janus kinase (JAK) and signal transducer and activator of transcription (STAT) DNA-binding proteins. Salas et al. reviewed the potential of JAK-STAT signaling for treating IBD [3]. Several factors activate JAK/STAT signaling, which controls the differentiation of M1 and M2 macrophages [4, 5]. The forkhead box O (FOXO) transcription factor family regulates cell proliferation and death, oxidative stress, inflammation, autophagy, and innate immune homeostasis [6], which can induce M1 macrophage polarization to inhibit interleukin-10 (IL-10) secretion [7].

IBD is associated with the decreased abundance and diversity of fecal bacteria, particularly short-chain fatty acid (SCFA)-producing bacteria, including *Roseburia hominis*, *Faecalibacterium prausnitzii*, *Bifidobacterium adolescentis*, and *Clostridium* cluster XIVa, and lower concentrations of organic acids [8–10]. SCFA-producing bacteria and their metabolites attenuate UC by modulating innate and acquired immunity in the mouse gut [11, 12]. For instance, *F. prausnitzii* secretes butyrate and other anti-inflammatory molecules to maintain immune homeostasis [13, 14]. *Akkermansia muciniphila* suppresses the expression of pro-inflammatory cytokines during chronic UC in mice [15]. A membrane protein from *A. muciniphila* improves UC by reducing macrophage infiltration and the number of CD8^+^ T cells [16]. Butyrate-producing *Clostridium butyricum* induces the synthesis of interleukin-10 (IL-10) by macrophages in inflamed mucosa [17].

The gut microbiota is strongly involved in the development of IBD. Our previous meta-analysis assessed the effects of fecal microbiota transplantation (FMT) into the lower gastrointestinal tract on UC and showed that a higher dose effectively induced remission [18]. However, patients with IBD, dysbiosis, and immunosuppression who receive FMT from healthy donors have an increased risk of developing bacteremia [19]. The inherent difficulty in determining the composition and stability of stool bacterial taxa limits their wider application [20]. Another approach to reduce inflammation is using a mixture of symbiotic bacteria [21]. Commonly recognized probiotics from the orders Lactobacillales, Clostridiales, and Verrucomicrobiales alleviate UC and improve outcomes [11, 16, 22]. In addition, the crosstalk of different strains is complex, and aerobic and facultative anaerobic bacteria consume oxygen in the distal intestine, favoring the colonization of strict anaerobes [23]. Therefore, members of these three orders are good candidates for the development of microbiome-based therapeutics.

Recent research in mice has focused on the treatment of UC using a single anaerobic bacterial strain. However, simulating normal intestinal microecology is essential to establish a dynamic and balanced system to produce stronger and longer-lasting therapeutic effects.

This study selected seven probiotic SCFA-producing strains, including strict and facultative anaerobes and aerobes, from the orders Lactobacillales, Verrucomicrobiales, and Clostridiales. The study assessed the efficacy of a mixture of these seven strains (7-mix), the resulting culture supernatant mixture (mix-sup), and FMT on UC in vivo and in vitro and discussed underlying mechanisms in colitic mice and human intestinal epithelial cell lines. The results demonstrate the potential of live SCFA-producing symbiotic bacterial strains and their products to treat intestinal diseases.

## Materials and methods

### Database analysis

The Inflammatory Bowel Disease Multi’omics Database (IBDMDB, https://ibdmdb.org/) is a comprehensive resource linking IBD to gut microbiota. This study, involving 107 subjects from the IBDMDB, explores differences in gut bacteria abundance between IBD patients (52 active CD, 21 active UC) and 34 healthy controls. Correlation analyses with disease activity were conducted using R version 4.2.0 and R Studio version 1.2.5001.

### Sample collection

Fresh fecal samples were acquired from 31 patients with active UC and six healthy donors from the Department of Gastroenterology and Hepatology at Guangzhou First People’s Hospital. Intestinal tissue inflammation in UC patients was assessed by endoscopy and histology. Patients had abstained from antibiotics, probiotics, and other medications for 3 months prior to sampling. Healthy donors were screened following a strict FMT protocol [24]. Samples were snap-frozen and stored at -80 °C.

### Sequencing

Total fecal DNA was extracted using the TIANamp Bacteria DNA Kit and analyzed by NovoGene (Beijing, China). The V3 − V4 region of the 16S rRNA gene was amplified by PCR and sequenced on an Illumina NovaSeq platform. Bioinformatics analyses involved operational taxonomic unit clustering [25] and measurement of α-diversity (Chao1 and Shannon indices) and β-diversity (principal coordinate analysis, PCoA) using QIIME software. Functional profiles were predicted from 16S rRNA data using Tax4Fun2.

### Strain isolation and culture

Fecal bacteria were isolated and cultured as described previously [26, 27]. Three SCFA-producing bacterial strains (FDAARGOS_771 [GenBank: CP053998.1], *Enterococcus hirae* ATCC 9790 [Taxonomy ID:768486], and *Ligilactobacillus salivarius* DSM 20555 [Taxonomy ID:1423799]) were isolated from healthy donors. *F. prausnitzii* A2-165 (DSM 17677) and *A. muciniphila* (DSM 22959) were purchased from Deutsche Sammlung von Mikroorganismen und Zellkulturen. *Lactobacillus casei* (ATCC 393) and *Clostridium butyricum* (ATCC 19398) were purchased from the American Type Culture Collection (ATCC). These cultures were combined to produce a 7-strain mixture (7-mix).

### Measurement of SCFA concentrations by liquid/gas chromatography‒mass spectrometry (LC-MS/GC-MS)

SCFA concentrations in bacterial culture supernatants were measured via liquid chromatography‒mass spectrometry (LC-MS). Mouse fecal SCFA concentrations were quantified by gas chromatography‒mass spectrometry (GC-MS) using a standard curve.

### Mouse model of acute ulcerative colitis (UC)

Thirty male BALB/c mice (age: 6–8 weeks) were purchased from Guangdong Medical Laboratory Animal Center (SCXK 2018-0002, Foshan, China). Mice were maintained under specific pathogen-free conditions with a 12-h light/12-h dark cycle, constant temperature (24 °C) and humidity (50%), and *ad libitum* access to standard chow and water. Then, 24 mice were fed 3% dextran sulfate sodium (DSS) (Sigma, USA) in drinking water for 7 days (once per day) to induce acute UC [28] and were randomly assigned to receive 7-mix, culture supernatant mixture (mix-sup), human FMT (hu-FMT), or phosphate-buffered saline (PBS) (0.1 mL per 10 g of body weight) intragastrically for 7 days (six animals per group). The disease activity index (DAI) was calculated based on weight loss, stool characteristics, and rectal bleeding [29].

### Preparation of bacterial strains and hu-FMT

Bacteria in the logarithmic growth phase were isolated. *E. hirae* and *L. casei* (aerobes) and *S. salivarius* (facultative anaerobe) were grown in MRS medium under aerobic conditions at 37 °C for 24–48 h. *F. prausnitzii*, *A. muciniphila*, *C. butyricum*, and *L. salivarius* (obligate anaerobes) were cultured under anaerobic conditions at 37 °C for 3–7 days. *F. prausnitzii* and *A. muciniphila* were grown in LYBHIv4 medium [30] and AKK medium, respectively (Supplementary Tables 1 and 2). *C. butyricum* and *L. salivarius* were grown in MRS medium. The seven strains were harvested and mixed in equal proportions (1.0 × 10^8^ CFU per strain). Each sample was centrifuged at 1500*g* for 5 min. The pellet was washed twice with PBS and suspended in 1 mL of sterile PBS. The culture supernatants were filtered through a 0.22 μm sterile filter. These two samples were labeled as 7-mix and mix-sup, respectively.

Fresh fecal samples (150–200 g) from a healthy 28-year-old woman were treated using an automatic purification system (GenFMTer, FMT Medical, Nanjing, China) as previously described [31]. Each sample was added to 1000 mL of sterile saline, and a bacterial suspension was prepared using the GenFMTer system at a centrifugation speed of 2000*g* for 5 min. The supernatant was discarded, and the pellet was resuspended in sterile saline. The final volume was completed to 200 mL with sterile saline, and the sample was labeled as hu-FMT.

### Macrophage depletion

Mice were injected intraperitoneally with 200 µL of clodronate (CLD) liposomes (Amsterdam, the Netherlands) to deplete macrophages. In the mouse model of acute UC, macrophages were depleted on days 4 and 1 before DSS treatment to deplete resident macrophages and on days 2, 5, and 7 after treatment to deplete new infiltrating macrophages [32]. DSS-treated mice injected with 200 µL of PBS served as controls.

### Cell culture

The mouse macrophage cell line RAW264.7, the human colorectal adenocarcinoma cell line Caco-2, and the human normal colon epithelial cell line NCM460 were obtained from ATCC. All cells were maintained in DMEM medium (Gibco, USA) with 10% fetal bovine serum (Procell, China) in a humidified atmosphere containing 5% CO_2_ at 37 ℃. RAW264.7 cells were seeded in a 24-well culture plate at a density of 5.0 × 10^5^ cells/mL and stimulated with 1 µg/mL lipopolysaccharide (LPS from *Escherichia coli* serotype 055:B5, Sigma‒Aldrich, USA) for 6 h to induce M1 macrophage polarization. To evaluate the effect of the STAT3 inhibitor S3I-201 (Abcam, UK) and the JAK2 inhibitor fedratinib (SAR302503, Selleck, China) on JAK/STAT3/FOXO3 signaling, cells were incubated with 7-mix (2^− 2^, namely 2.5 × 10^7^ CFU), mix-sup (2^− 4^), S3I-201 (50 µM), fedratinib (10 µM), or S3I-201 + fedratinib for 4 h.

### Transwell assay

Cell interactions were assessed using Caco-2/RAW264.7 and NCM460/RAW264.7 transwell co-culture systems. In brief, Caco-2 and NCM460 cells in DMEM medium were transferred to the lower chamber of a 24-well transwell (5.0 × 10^5^ cells/well). Then, cells were treated with 1 µg/mL LPS and incubated in a humidified incubator with 5% CO_2_ at 37 °C for 24 h. RAW264.7 cells were seeded in 100 µL of DMEM medium as a monolayer on a polycarbonate membrane in transwell inserts (0.33 cm^2^, 0.4 μm pore size; Corning Costar Corp.) and were treated with 7-mix (2^− 2^) or mix-sup (2^− 4^) in the lower chamber of a transwell for 4 h. Untreated RAW264.7 cells were used as controls. RAW264.7 cells treated with 7-mix or mix-sup were washed twice with sterile PBS and co-cultured with LPS-treated epithelial cells for 24 h in another transwell system. Caco-2 and NCM460 cells were harvested for further analysis.

### Quantitative reverse transcription-polymerase chain reaction (qRT-PCR)

Total RNA was extracted from the colon tissues of mice or cultured cells using TRIzol Reagent. RNA was reverse-transcribed to cDNA using the PrimeScript™ RT reagent Kit (TaKaRa, Japan) according to the manufacturer’s protocol. PCR was performed using the SYBR Green Premix Pro Taq HS qPCR Kit (Accurate Biology, China). The primer sequences are listed in Supplementary Table 3.

### Histopathology

Mouse colon tissue was harvested and snap-frozen in liquid nitrogen. The tissue was washed twice with sterile PBS, and approximately 1.0 cm of tissue was fixed in 4% formaldehyde and embedded in paraffin. Paraffin-embedded Sect. (4 μm) were stained with hematoxylin and eosin (HE). Tissue damage was scored histologically by two independent investigators using a semi-quantitative method as follows: epithelium loss (0, none; 1, within the basal 1/3; 2, in the basal 1/3 to 2/3; 3, over the basal 2/3), crypt damage (0, none; 1, less than 1/3; 2, 1/3 − 2/3; 3, more than 2/3), and inflammatory cell infiltration (0, none; 1, mild; 2, moderate; and 3, severe) [28]. The three parameters were summed to generate a maximum score of 9.

### Immunohistochemistry

Intestinal barrier function was assessed by measuring the protein expression of Muc2, ZO-1, and occludin in colon samples. The degree of acute inflammatory cell infiltration was evaluated by measuring MPO expression. The protein expression of STAT3 and FOXO3 was also measured. Paraffin-embedded mouse colon samples were labeled with rabbit anti-MUC2 antibody (1/2000; Abcam, ab272692), ZO-1 (1/100; Abcam, ab216880), occludin (1/100; Abcam, ab168986), MPO (1/1000 dilution; Abcam, ab208670), STAT3 (1/1200 dilution; ABclonal, A19566), and FOXO3 (1/1000; CST, 2497 S), followed by incubation with goat anti-rabbit horseradish peroxidase-conjugated polyclonal secondary antibody (ab214880) for 30 min at room temperature. Immunostaining was performed using the DAB Detection Kit (Gene Tech, Shanghai, China). Protein expression was scored by two independent researchers based on the percentage of positively-stained cells (0, < 5%; 1, 6–25%; 2, 26–50%; 3, 51–75%; and 4, 76–100%) and staining intensity (0, none; 1, mild; 2, moderate; and 3, strong) [31]. The total score was the product of staining extent and intensity scores. A score of 6 was the threshold for high protein expression levels.

### Immunofluorescence staining

Colon tissue Sect. (4 μm) were incubated with rabbit F4/80 antibody (1/400; CST, 30325T) or CD206 antibody (1/200; CST, 24595T) at 4 ℃ overnight. Then, tissue samples were incubated with fluorescent secondary antibodies—IgG conjugated to the Alexa Fluor 488 F(ab’)_2_ fragment of anti-rabbit IgG (H + L), or IgG conjugated to the Alexa Fluor 555 F(ab’)_2_ fragment of anti-rabbit IgG (H + L)—in the dark for 30 min. Nuclei were stained with DAPI (Beyotime, China) and an anti-fluorescence quencher. Tissue sections were imaged using a laser-scanning confocal microscope (Nikon A1HD25).

### Flow cytometry analysis

Mouse colon tissues were digested with 0.5% collagenase IV and 0.5% DNase I. Then, colonic cells were stained with fluorescein isothiocyanate-conjugated anti-mouse CD45 (BioLegend, 157608), allophycocyanin (APC)/cyanine (Cy) 7-conjugated anti-mouse CD3 (BioLegend, 100221), BB700 anti-mouse CD4 (BD Biosciences, 566407), APC anti-mouse CD25 (BioLegend, 101909), APC/Cy 7 anti-mouse/human CD11b (BioLegend, 101226), phycoerythrin (PE) anti-mouse F4/80 (BioLegend, 123109), APC anti-mouse CD86 (BioLegend, 105011), Brilliant Violet 421 anti-mouse CD80 (BioLegend, 104725), and Brilliant Violet 421 anti-mouse CD206 (BioLegend, 141717) for cell surface staining. Cells were permeabilized with intracellular staining perm wash buffer (BioLegend, 421002) and stained with Brilliant Violet 42 anti-mouse T-bet (BioLegend, 644815), PE anti-mouse GATA3 (BioLegend, 653803), PE anti-mouse Foxp3 (BioLegend, 126403), and Brilliant Violet 421 anti-mouse RORγt (BD Biosciences, 562894). Samples were analyzed on a FACS Canto II cytometer (BD Biosciences), and data were analyzed using FlowJo (TreeStar, USA). Data on adherent and dead cells were processed and gated on viable single cells. Gating strategies are shown in Supplementary Fig. 1A-C. Gates for nuclear transcription factors (GATA3 and FOXP3) were set based on Fluorescence Minus One controls. Quantitative data are shown as mean ± standard deviation (SD).

### Western blotting

Total protein was extracted using RIPA buffer containing protease and phosphatase inhibitors, and protein concentration was measured using a bicinchoninic acid assay, and the manufacturer allowed other research groups to replicate the methods. Proteins were separated by SDS‒PAGE and transferred onto polyvinylidene difluoride membranes. The membranes were incubated with rabbit anti-JAK2 (ABclonal, A19629), anti-pJAK2 (ABclonal, AP0531), anti-FOXO3 (CST, 2497 S), anti-STAT3 (ABclonal, A19566), and anti-pSTAT3 (ABclonal, AP0705) at a 1/1000 dilution overnight at 4 °C, followed by incubation with goat anti-rabbit secondary antibody (ab214880) for 30 min at room temperature. Immunoreactive bands were visualized using an enhanced chemiluminescence detection system. Fluorescence intensity was quantified using ImageJ. The experiments were repeated thrice.

### Statistical analysis

The experiments were performed independently at least three times. Continuous variables are expressed as means ± SDs. Statistical analysis was performed using SPSS Statistics software version 24.0 (IBM). Pairwise comparisons were performed using Student’s *t*-test, and comparisons between three or more groups were performed using one-way analysis of variance. P-values of less than 0.05 were considered statistically significant.

## Results

### The abundance of SCFA-producing bacteria is markedly decreased in patients with active UC

**Fig. 1:**
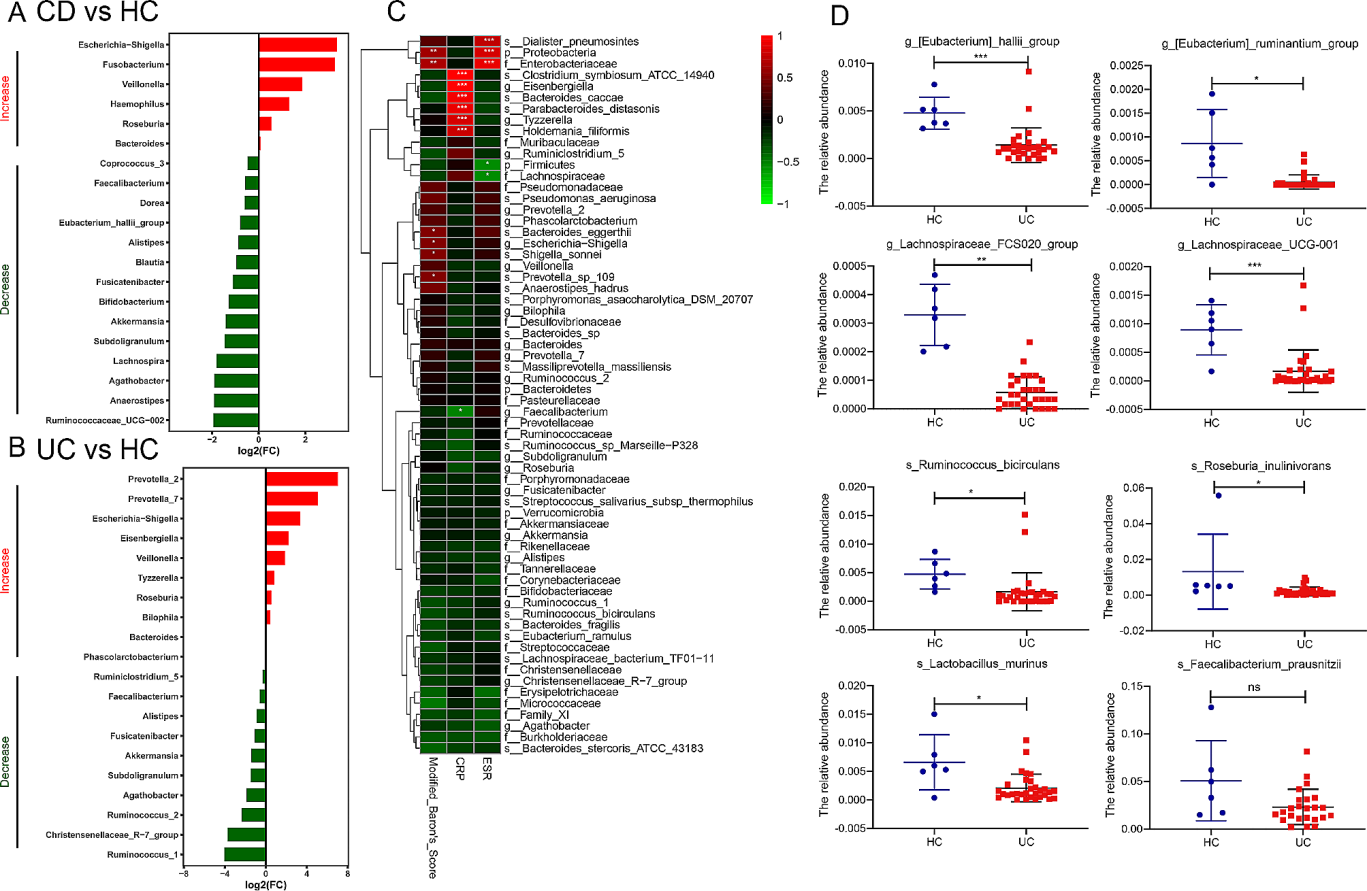
The abundance of short-chain fatty acid (SCFA)-producing bacteria is markedly decreased in patients with active UC. **A-B**. Abundance of short-chain fatty acid (SCFA)-producing bacteria in patients with active Crohn’s disease (CD) (*n* = 52) and ulcerative colitis (UC) (*n* = 21) compared with healthy controls (*n* = 34) from the Inflammatory Bowel Disease Multi’omics Database. **C**. Correlation analysis between microbial abundance and inflammatory markers of UC (modified Baron’s score, C-reaction protein [CRP], and erythrocyte sedimentation rate [ESR]). **D**. Relative abundance of SCFA-producing bacteria in patients with active UC (*n* = 31) and healthy controls (HC) from Guangzhou First People’s Hospital (*n* = 6). *P < 0.05, **P < 0.01, ***P < 0.001 (Student’s *t*-test); ns, not significant

Alpha diversity (Chao1 index) and β-diversity (PCoA) were lower in the feces of patients with IBD than in those of healthy subjects from the IBDMDB database (Supplementary Fig. 2A-C). The abundance of microbial taxa was measured in these two groups, and log_2_ fold changes greater than 2.0 were considered significant. The relative abundance of SCFA-producing bacteria *Faecalibacterium*, *Eubacterium_hallii_group*, *Bifidobacterium*, *Akkermansia*, *Lachnospira*, *Ruminococcaceae*, *Lactobacillus_gasseri*, and *Ruminiclostridium* was lower in patients with active CD and UC than in healthy controls (Fig. 1A-B). Correlation analysis of microbial abundance and inflammatory markers of UC—modified Baron’s score, C-reaction protein level, and erythrocyte sedimentation rate—showed that inflammatory conditions were associated with a higher abundance of some taxa and a decrease in the abundance of SCFA-producing bacteria (Supplementary Table 4).

There were moderate and strong positive correlations between acute UC and the abundance of Proteobacteria, *Escherichia-Shigella*, *Shigella sonnei*, *Bacteroides eggerthii*, and *Parabacteroides distasonis* and weak and moderate negative correlations between acute UC and the abundance of Firmicutes, Lachnospiraceae, *Faecalibacterium*, Akkermansiaceae, Ruminococcaceae, Bifidobacteriaceae, and *Roseburia* (Fig. 1C). Moreover, the relative abundance of SCFA-producing bacteria was lower in 31 patients with active UC than in six healthy individuals from our research center (Fig. 1D). These results indicate that the abundance of SCFA-producing bacteria was reduced in UC.

### Seven-mix and mix-sup improved DSS-induced acute UC and gut barrier function

**Fig. 2:**
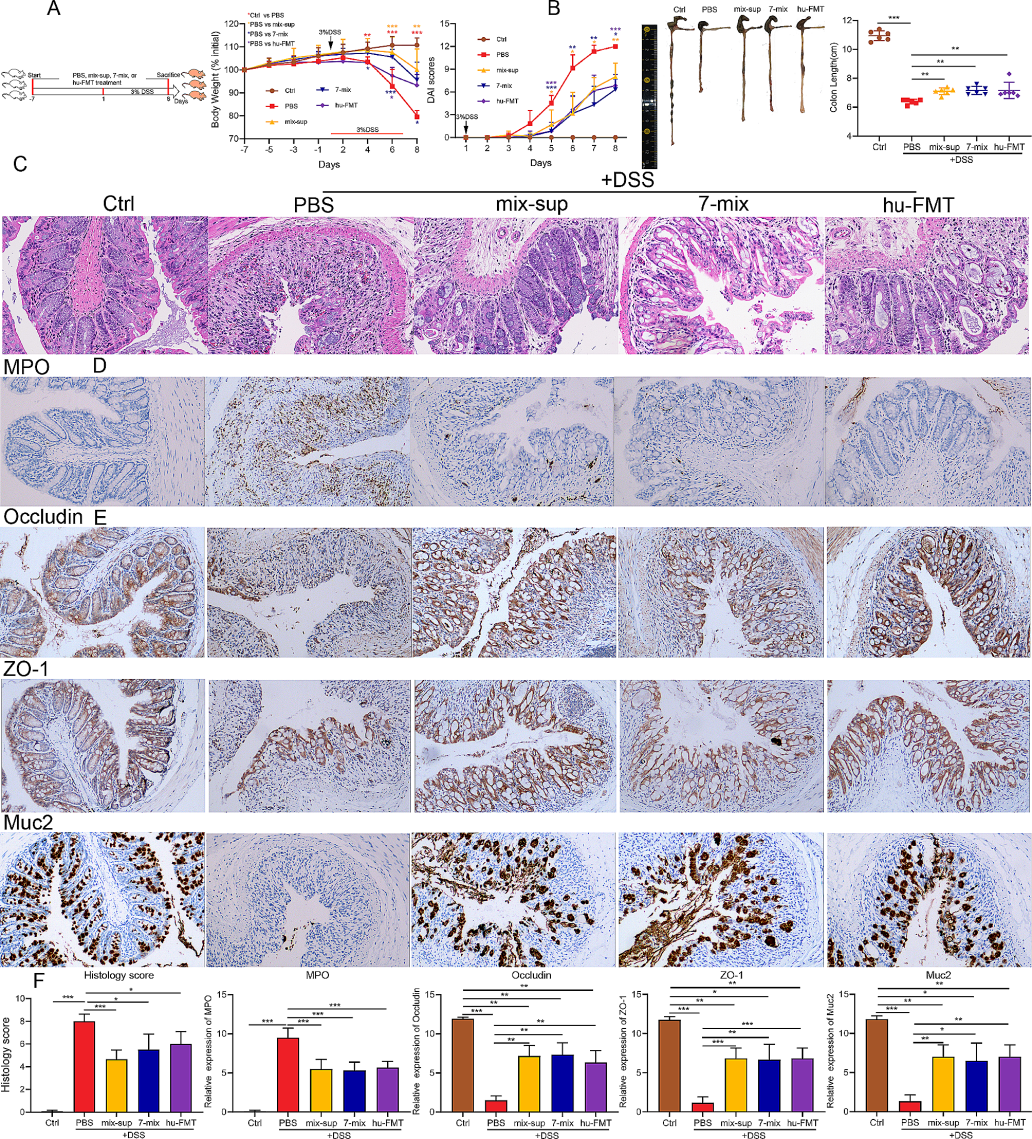
Treatment with a mixture of seven short-chain fatty acid-producing bacterial strains (7-mix) and the resulting culture supernatant mixture (mix-sup) reduces acute ulcerative colitis (UC) and improves gut barrier function. **A**. Experimental mouse model of acute UC. **B**. Body weight, disease activity index (DAI) scores, and colon length in colitic mice treated with PBS, 7-mix, mix-sup, or fecal microbiota transplantation (FMT) relative to control mice. **C-E**, Colon tissue morphology (hematoxylin-eosin staining, ×200) and the immunohistochemical expression of myeloperoxidase (MPO), occludin, ZO-1, and Muc2 (×200) in colitic mice treated with PBS, 7-mix, mix-sup, or FMT relative to control mice. **F**, Histological scores and semi-quantitative analysis of immunohistochemical staining. *N* = 5–6. *P < 0.05, **P < 0.01, ***P < 0.001

To clarify the role of 7-mix and mix-sup in UC, we fed mice 3% DSS in drinking water for 7 days to induce acute UC and assessed the effect of 7-mix, mix-sup, hu-FMT, or PBS on UC (Fig. 2A). The ability of bacteria to produce SCFAs was analyzed by LC-MS (Supplementary Table 5). All strains produced SCFAs, although the type and amount differed across strains. We observed that 7-mix, mix-sup, and hu-FMT were associated with less body weight loss, lower DAI scores, and longer colon length than PBS (Fig. 2B). In addition, 7-mix, mix-sup, and hu-FMT decreased colonic inflammation, manifested by improved crypt structure and less inflammatory cell infiltration (Fig. 2C-D, F). These results showed that 7-mix, mix-sup, and hu-FMT alleviated acute UC in mice. However, there was no significant difference in the effectiveness of these three interventions.

Next, we examined the effect of 7-mix and mix-sup on intestinal barrier function in a mouse model of UC. Immunohistochemistry (IHC) and qRT-PCR analysis of occludin and ZO-1 (tight junction markers) and MUC2 (a marker of goblet cell metaplasia) revealed that 7-mix, mix-sup, and hu-FMT improved gut barrier function (Fig. 2E-F, Supplementary Fig. 3). These data demonstrate that treatment with 7-mix and mix-sup improved gut barrier integrity.

#### Seven-mix and mix-sup induced M2 macrophage polarization

**Fig. 3:**
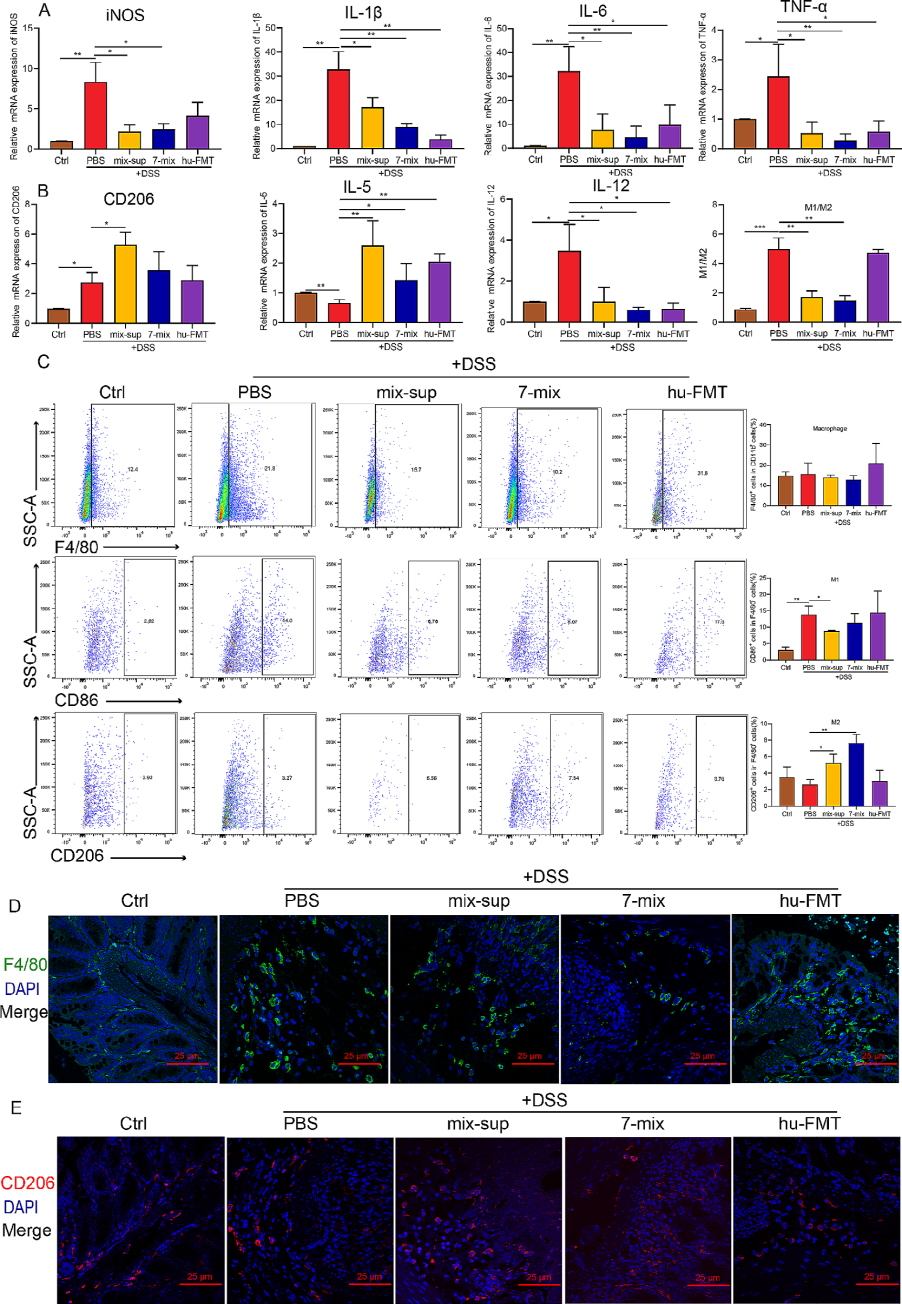
Treatment with a mixture of seven short-chain fatty acid-producing bacterial strains (7-mix) and the resulting culture supernatant mixture (mix-sup) regulates macrophage polarization. **A-B**. qRT-PCR analysis of the effect of 7-mix, mix-sup, and fecal microbiota transplantation (FMT) on the mRNA expression of cytokines produced by M1 and M2 macrophages. **C**. Flow cytometry analysis of the effect of 7-mix, mix-sup, and FMT on macrophage polarization. **D-E**. Immunofluorescence analysis of the effect of 7-mix, mix-sup, and FMT on the number of F4/80^+^ and CD206^+^ macrophages (×400). *N* = 3–4. *P < 0.05, **P < 0.01, ***P < 0.001

To investigate whether 7-mix and mix-sup improved innate and acquired immunity in mice with acute UC, the mRNA expression of inflammatory factors in the inflamed colon was measured by qRT-PCR. The qRT-PCR and flow cytometry results showed that 7-mix and mix-sup upregulated the expression of cytokines produced by Th2 cells (GATA3, IL-4) and Treg cells (FOXP3, IL-10); in turn, hu-FMT downregulated the expression of inflammatory cytokines produced by Th1 cells (T-bet, IFN-γ) and Th17 cells (retinoic acid receptor-related orphan receptor gamma t [RORγt], IL-17 A) (Supplementary Fig. 4A-E). Given the critical role of macrophages in intestinal immunity, the effects of treatment on intestinal macrophage polarization were evaluated. The qRT-PCR results showed that 7-mix, mix-sup, and hu-FMT decreased the levels of the M1-associated pro-inflammatory cytokines iNOS, IL-1β, IL-6, TNF-α, and IL-12 (Fig. 3A) and increased the expression of two M2-related anti-inflammatory cytokines: CD206 and IL-5 (Fig. 3B).

Flow cytometry data confirmed that 7-mix and mix-sup decreased the number of M1 (CD86^+^) macrophages and increased the number of M2 (CD206^+^) macrophages. Furthermore, the M1/M2 ratio indicated that 7-mix and mix-sup exerted an anti-inflammatory effect by regulating macrophage polarization in the mouse colon. However, hu-FMT had no such effects (Fig. 3C). Immunofluorescence staining showed that 7-mix, mix-sup, hu-FMT, and PBS increased the number of macrophages (F4/80) to varying degrees (Fig. 3D), whereas the M2 phenotype (CD206^+^) was dominant in the mix-sup and 7-mix treatment groups (Fig. 3E). In summary, 7-mix and mix-sup achieved beneficial effects by regulating innate immunity via macrophage activation and polarization in a mouse model of acute UC.

### Seven-mix and mix-sup increased the abundance of SCFA-producing bacteria and SCFA concentrations

**Fig. 4:**
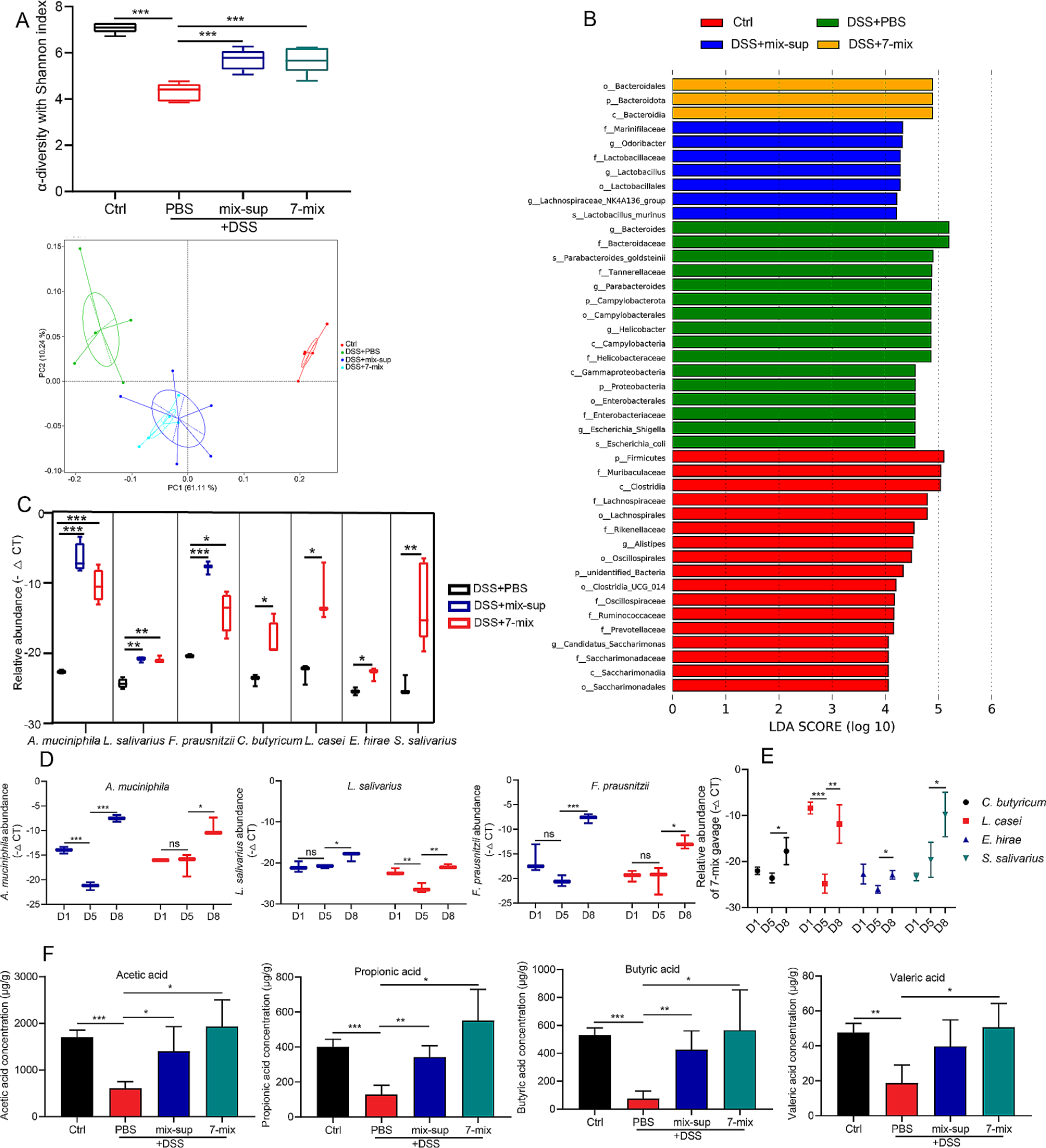
Effect of a mixture of seven short-chain fatty acid (SCFA)-producing bacterial strains (7-mix) and the corresponding culture supernatant mixture (mix-sup) on bacterial diversity and abundance and SCFA concentrations in the feces of colitic mice. **A**. α-diversity (Shannon index) and β-diversity (principal coordinate analysis) in the feces of colitic mice treated with PBS, 7-mix, or mix-sup. **B**, Linear discriminant analysis (LDA) effect size analysis of differentially abundant (LDA scores > 4) taxa in colitic mice treated with PBS, 7-mix, or mix-sup. **C**. qRT-PCR analysis of the relative abundance of seven bacterial species in the feces of colitic mice treated with PBS, 7-mix, or mix-sup at the experimental endpoint. **D.** Relative abundance of three bacterial strains in the mix-sup group (blue bars) and 7-mix group (red bars) on days 1, 5 and 8 after colitis induction. **E**. Relative abundance of four bacterial strains in the 7-mix group on days 1, 5, and 8 after colitis induction. **F**, SCFA levels in the study groups. *N* = 3–5, *P < 0.05, **P < 0.01, ***P < 0.001

We next investigated the effect of 7-mix and mix-sup on gut bacterial diversity and abundance using 16S rRNA gene sequencing. Compared with PBS, 7-mix and mix-sup significantly increased α-diversity (Shannon index) and β-diversity (PCoA) in the feces of colitic mice (Fig. 4A). Moreover, linear discriminant analysis effect size showed an increase in the relative abundance of g_*Bacteroides*, g_*Parabacteroides*, g_*Helicobacter*, p_Proteobacteria, and s_*Escherichia coli* in the UC group. However, we found an increase in the relative abundance of f_Lactobacillaceae, g_*Lactobacillus*, g_*Lachnospiraceae*, and s_*Lactobacillus murinus* in the mix-sup group and an increase in the relative abundance of o_Bacteroidales in the 7-mix group (Fig. 4B). The qRT-PCR results at the experimental end point indicated that mix-sup increased the abundance of *A. muciniphila*, *L. salivarius*, and *F. prausnitzii*, and 7-mix increased the abundance of *A. muciniphila*, *L. salivarius*, *F. prausnitzii*, *C. butyricum*, *L. casei*, *E. hirae*, and *S. salivarius* compared with PBS (Fig. 4C). To further investigate the effect of 7-mix and mix-sup on microbial colonization in the mouse gut, we collected feces on days 1, 5, and 8 after UC induction and found that the relative abundance of *A. muciniphila* and *F. prausnitzii* decreased on day 5 after induction, and mix-sup treatment for 7 days significantly increased the abundance of *A. muciniphila*, *L. salivarius*, and *F. prausnitzii* on day 8 (Fig. 4D). An explanation for this result is that mix-sup (primarily SCFAs) provides raw materials for the proliferation of SCFA-producing bacteria. In addition, the abundance of *L. salivarius* and *L. casei* decreased on day 5 after induction, and 7-mix treatment for 1 week enhanced the colonization of the seven strains (Fig. 4D-E).

SCFA levels in the mouse stool were measured by GC-MS. The results indicated that 7-mix and mix-sup significantly increased the concentrations (µg/g) of acetic, propionic, butyric, and valeric acid compared with PBS (Fig. 4F). These results demonstrate that DSS induced acute UC and dysbiosis and that 7-mix and mix-sup remodeled the gut microbiota and substantially increased SCFA concentrations in the gut.

### Seven-mix and mix-sup alleviated UC after macrophage depletion

**Fig. 5:**
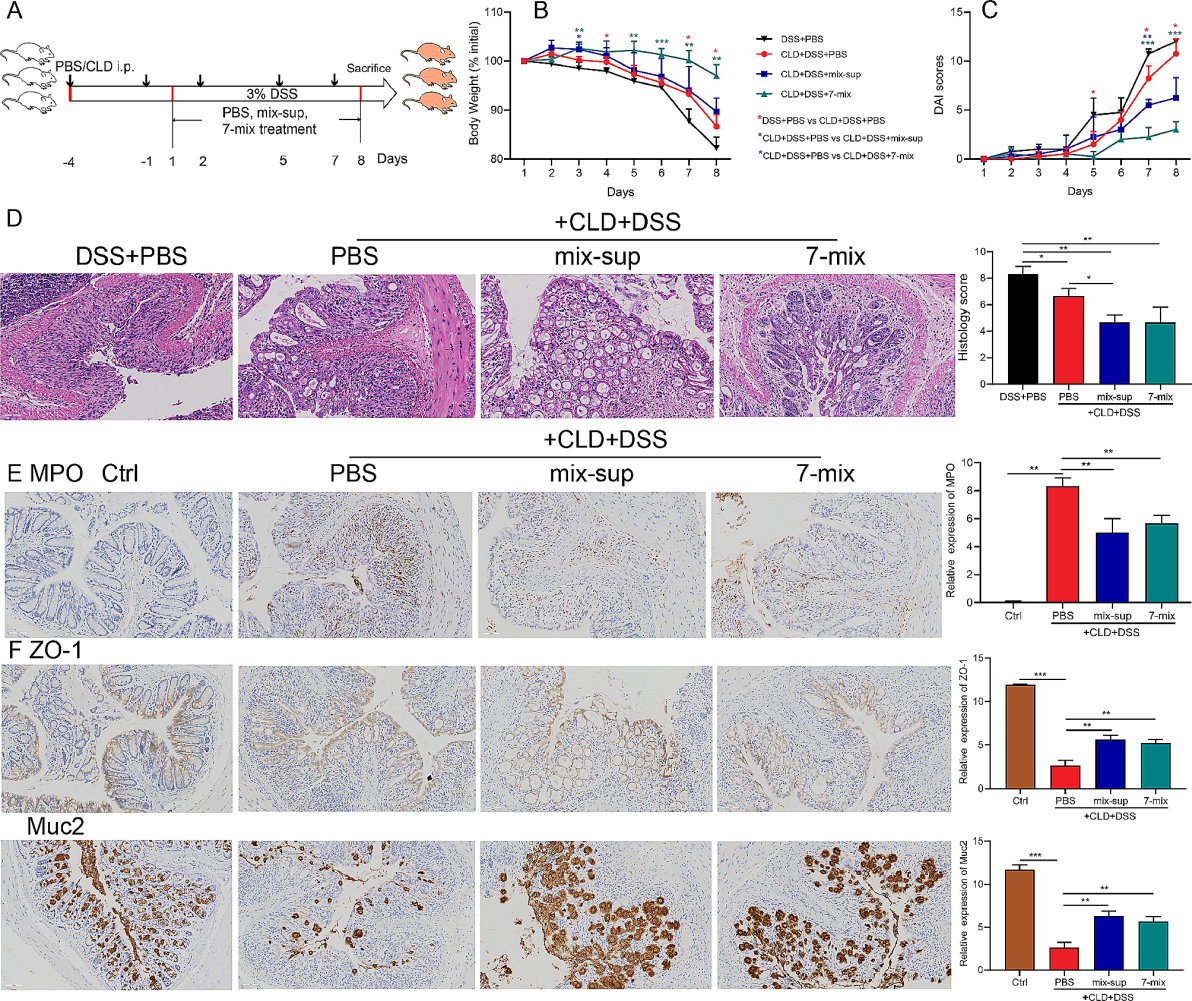
Treatment with a mixture of seven short-chain fatty acid-producing bacterial strains (7-mix) and the corresponding culture supernatant mixture (mix-sup) alleviates colitis after macrophage depletion in colitic mice. **A**. Macrophages were depleted by intraperitoneally injecting clodronate (CLD) liposomes in mice. **B-D**. Body weight, disease activity index (DAI) scores, and histological scores (×200) in colitic mice treated with PBS, 7-mix, or mix-sup and treated or not with CLD. **E-F**. Immunohistochemical expression of myeloperoxidase (MPO), ZO-1, and Muc2 in colitic mice treated with PBS, 7-mix or mix-sup and treated with CLD (×200). *N* = 3–5. *P < 0.05, **P < 0.01, ***P < 0.001

To investigate the role of macrophages in UC, mice were injected intraperitoneally (i.p.) with 200 µL of clodronate (CLD) liposomes to deplete macrophages (Fig. 5A). The results showed that CLD combined with 3% DSS decreased the number of macrophages in the spleen 24 h after injection compared with PBS; however, macrophage depletion was less pronounced in the colon than in the spleen (Supplementary Fig. 5). CLD treatment improved UC compared with PBS, manifested by less body weight loss and lower DAI scores (Fig. 5B-C), consistent with the literature [33, 34]. Moreover, 7-mix and mix-sup protected CLD-treated colitic mice by reducing colonic damage (Fig. 5D) and neutrophil infiltration (Fig. 5E) and improving intestinal barrier function (Fig. 5F) after spleen macrophage depletion. These data confirm that the beneficial effects of 7-mix and mix-sup do not depend on spleen macrophages.

### Seven-mix and mix-sup reduced UC and improved gut barrier function by promoting M2 macrophage polarization

**Fig. 6:**
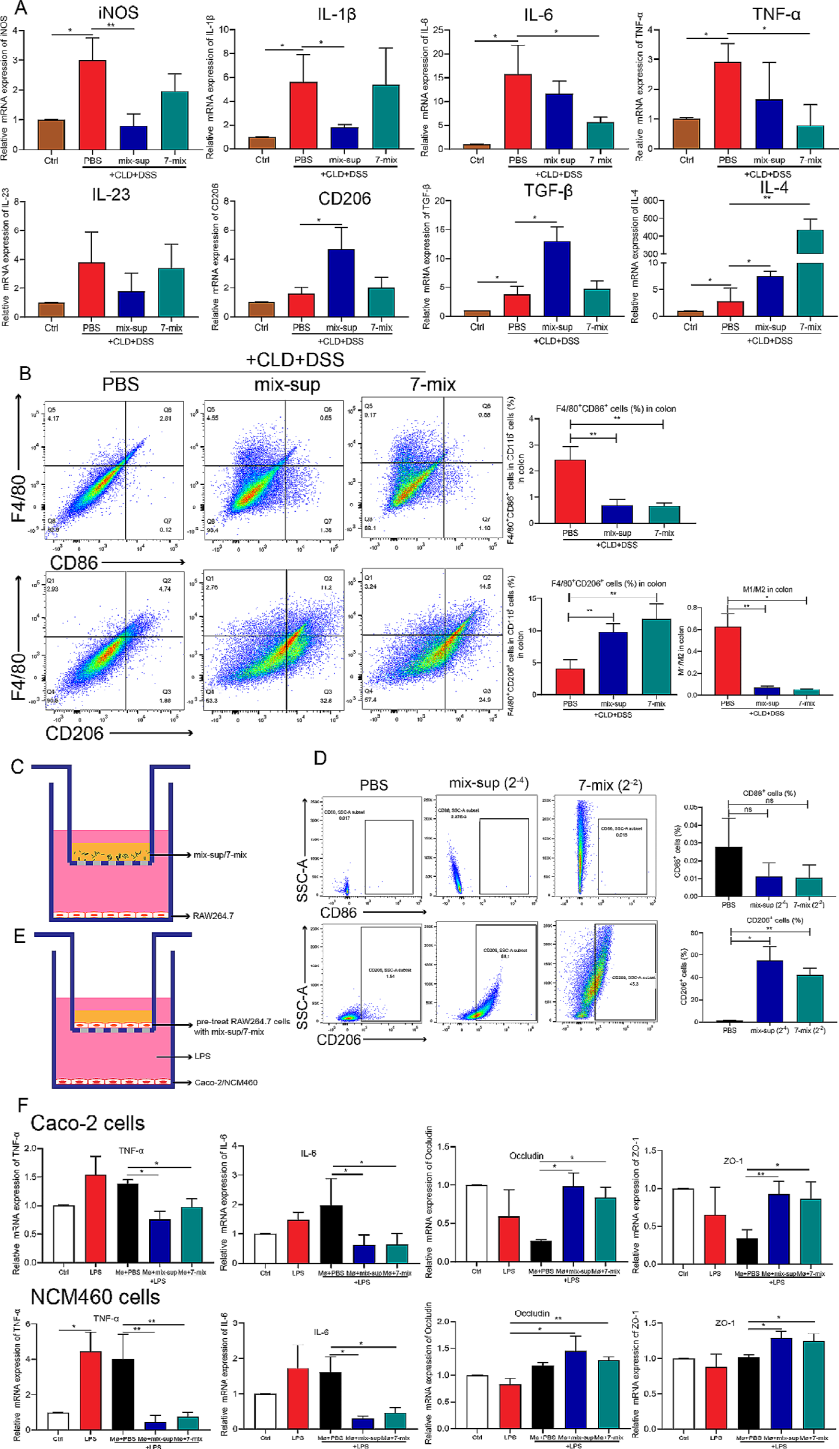
Treatment with a mixture of seven short-chain fatty acid-producing bacterial strains (7-mix) and the resulting culture supernatant mixture (mix-sup) induces M2 macrophage polarization, reducing ulcerative colitis and improving gut barrier function in macrophage-depleted mice. **A**. qRT-PCR analysis of the effect of 7-mix and mix-sup on the mRNA expression of cytokines produced by M1 and M2 macrophages. **B**. Flow cytometry analysis of the effect of 7-mix and mix-sup on macrophage polarization. **C**. RAW264.7 cells co-cultured with 7-mix and mix-sup. **D**. 7-mix and mix-sup induced M2 macrophage polarization. **E**. Caco-2/RAW264.7 and NCM460/RAW264.7 transwell co-culture system. **F**. qRT-PCR analysis of the effect of macrophage treatment with 7-mix or mix-sup on the mRNA expression of TNF-α, IL-6, occludin and ZO-1 in Caco-2 and NCM460 cells. *N* = 3–4. *P < 0.05, **P < 0.01, ***P < 0.001

After spleen macrophage depletion, 7-mix and mix-sup decreased the levels of M1-associated pro-inflammatory cytokines (iNOS, IL-1β, IL-6, TNF-α, and IL-23) and increased the levels of M2-related anti-inflammatory cytokines (CD206, TGF-β, and IL-4) in colon tissues of CLD-treated colitic mice (Fig. 6A). Moreover, flow cytometry results showed that 7-mix and mix-sup significantly decreased the number of M1 macrophages and promoted M2 macrophage polarization in the colon. Moreover, the ratio of M1/M2 macrophages indicated that 7-mix and mix-sup exerted anti-inflammatory effects by modulating macrophage polarization in the colon (Fig. 6B). However, these two interventions failed to stimulate spleen macrophages (Supplementary Fig. 6). These results indicate that the intragastric administration of 7-mix and mix-sup reduced UC by inhibiting intestinal M1 macrophage polarization after spleen macrophage depletion and promoting intestinal M2 macrophage polarization.

To confirm the effect of 7-mix and mix-sup on macrophage polarization in vitro, RAW264.7 cells were seeded into the lower chamber of a 24-well transwell at a density of 5.0 × 10^5^ cells/well, and 7-mix (2^− 2^) or mix-sup (2^− 4^) was added to transwell inserts for 4 h (Fig. 6C). The effect of 7-mix and mix-sup on M2 macrophage polarization was stronger than that of PBS (Fig. 6D). The effects of macrophages treated with 7-mix or mix-sup on intestinal epithelial cells were assessed using Caco-2/RAW264.7 and NCM460/RAW264.7 transwell co-cultures (Fig. 6E). Inflammation was induced in Caco-2 and NCM460 cells using LPS. Macrophage treatment with 7-mix and mix-sup significantly downregulated TNF-α and IL-6 and upregulated occludin and ZO-1 in Caco-2 and NCM460 cells (Fig. 6F). These data confirmed that these treatments improved UC and gut barrier function by directly inducing M2 macrophage polarization.

### Seven-mix and mix-sup inhibited JAK/STAT3/FOXO3 pathway activation in macrophages

**Fig. 7:**
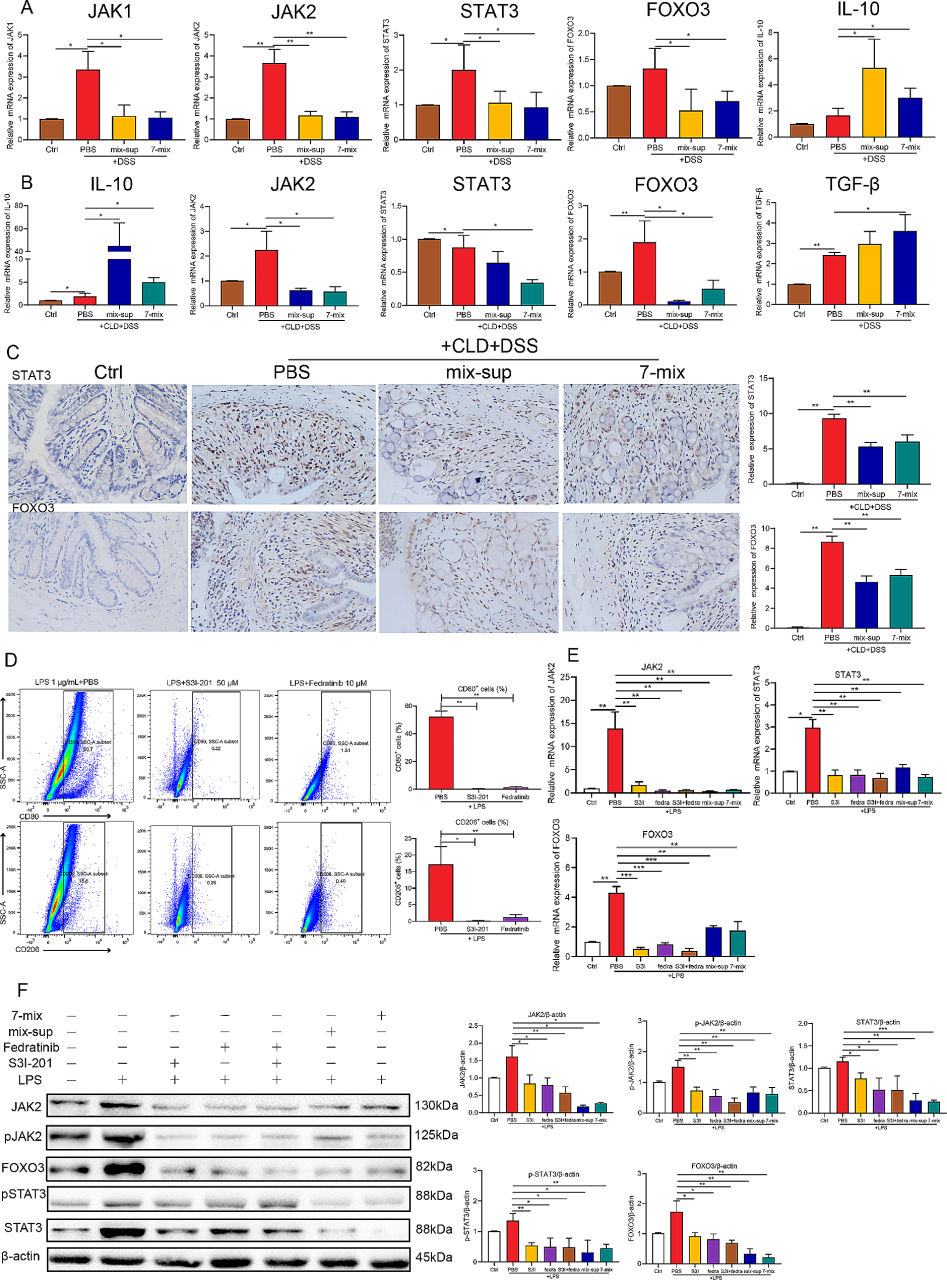
Treatment with a mixture of seven short-chain fatty acid-producing bacterial strains (7-mix) and the resulting culture supernatant mixture (mix-sup) inhibits JAK/STAT3/FOXO3 pathway activation in macrophages. **A-B**. qRT-PCR analysis of the effect of 7-mix and mix-sup on the regulation of the JAK/STAT3/FOXO3 axis in colitic mice treated or not with clodronate (CLD) liposomes. **C**. Effect of 7-mix and mix-sup on the immunohistochemical expression of STAT3 and FOXO3 in colitic mice treated with CLD. **D**. Flow cytometry analysis of the effect of S3I-201 and fedratinib on macrophage polarization. **E**. qRT-PCR analysis of the effect of 7-mix, mix-sup, S3I-201, and fedratinib on the mRNA expression of JAK2, STAT3, and FOXO3 in LPS-treated RAW264.7 cells. **F**. Western blot analysis of the effect of 7-mix, mix-sup, S3I-201, and fedratinib on the protein expression of JAK2, p-JAK2, STAT3, p-STAT3, and FOXO3 in LPS-treated RAW264.7 cells. *N* = 3. *P < 0.05, **P < 0.01, ***P < 0.001

**Fig. 8:**
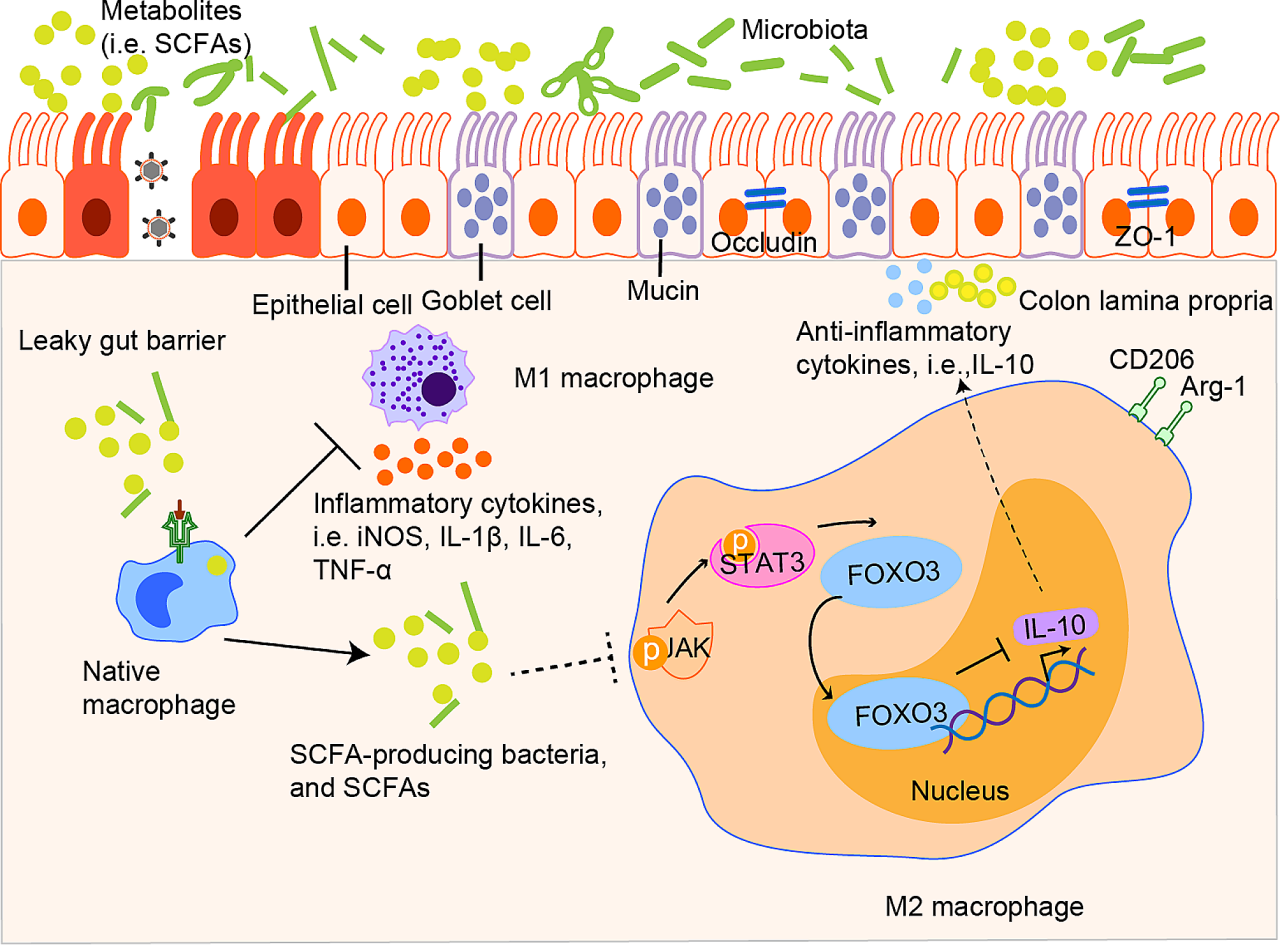
Schematic representation of the regulation of dextran sulfate sodium-induced colitis by short-chain fatty acid (SCFA)-producing bacterial strains and SCFAs. Colitis damages the gut barrier, inducing the activation of M1 macrophages, which produce pro-inflammatory cytokines, including IL-1β, IL-6, and TNF-α. In turn, SCFA-producing bacterial strains and SCFAs stimulate the activation of M2 macrophages, which produce anti-inflammatory cytokines (i.e., IL-10) to inhibit JAK/STAT3/FOXO3 axis activation and improve gut barrier function

Salas et al. evaluated the role of JAK-STAT signaling in intestinal homeostasis and pathological processes and the therapeutic potential of this pathway in IBD [3]. The transcription factor FOXO3 inhibits IL-10 secretion by inducing M1 macrophage polarization [7]. We examined the contribution of 7-mix and mix-sup to JAK/STAT3/FOXO3 phosphorylation in macrophages. The qRT-PCR results revealed that DSS-induced UC upregulated JAK2, STAT3, and FOXO3 in mice treated or not with CLD, and 7-mix and mix-sup reversed this effect. In contrast, 7-mix and mix-sup enhanced the effects of UC on IL-10 expression in mice treated or not with CLD and enhanced the effect of UC on TGF-β expression (Fig. 7A-B). The results on STAT3 and FOXO3 expression were corroborated by IHC (Fig. 7C) (See Fig. 8).

The hypothesis that these treatments improved gut barrier function via the JAK/STAT3/FOXO3 pathway in macrophages was tested using a STAT3 inhibitor (S3I-201) and a JAK2 inhibitor (fedratinib). Treatment of RAW264.7 cells with LPS (1 µg/mL) for 6 h increased the number of M1 (CD80^+^) and M2 (CD206^+^) macrophages, whereas S3I-201 and fedratinib reversed this effect (Fig. 7D). LPS upregulated JAK2, STAT3, and FOXO3, while S3I-201, fedratinib, S3I-201 plus fedratinib, 7-mix, and mix-sup reversed these effects (Fig. 7E). Moreover, western blots showed that 7-mix, mix-sup, fedratinib, and S3I-201 decreased the protein expression of JAK2, p-JAK2, STAT3, p-STAT3, and FOXO3 (Fig. 7F). These results indicated that 7-mix and mix-sup had protective roles in vivo and in vitro by inhibiting JAK/STAT3/FOXO3 axis activation in macrophages.

## Discussion

IBD is characterized by dysbiosis, immune dysfunction, and genetic susceptibility resulting in an abnormal gut barrier. FMT has good potential to treat IBD. However, changes in gut microbial diversity and stability in donors over time are difficult to predict. Furthermore, there are concerns about transferring potential pathogens to patients with dysbiosis and immunosuppression, increasing the risk of serious adverse events associated with FMT [35]. Strategies to restore the intestinal microbiota using specific bacterial species are urgently required. The advantages of these strategies include the selection of specific strains and the isolation and purification of their metabolites. In the present study, we selected seven probiotic bacteria at a 1:1 ratio and assessed the ability of these bacteria to produce SCFAs, such as acetate, propionate, and butyrate.

Our results showed that macrophages activated by 7-mix and mix-sup in the colon lamina propria, but not in the spleen, improved UC. To confirm this finding, macrophages were depleted by intraperitoneally injecting CLD liposomes in mice. Macrophage depletion was more pronounced in the spleen than in the colon. One explanation for this result is that intraperitoneally injected agents enter the bloodstream through the lymphatic system to deplete macrophages in tissues, including the colon. Given that lymph circulation is slower and less dynamic than blood circulation, macrophage depletion is associated with a slower onset and shorter duration in the colon than in the spleen.

CLD decreased inflammation during UC, consistent with the literature [33, 34]. Furthermore, 7-mix and mix-sup reduced colonic damage and improved intestinal barrier function in the presence of spleen macrophage depletion. We explored whether these treatments stimulated macrophage polarization after spleen macrophage depletion and found that these interventions activated macrophages in the colon lamina propria rather than in the spleen in vivo. The effects of 7-mix and mix-sup on M2 macrophage polarization were evaluated in vitro using a transwell co-culture system and two colon epithelial cell lines. Seven-mix and mix-sup attenuated UC and leaky gut by reducing M1 macrophage polarization and promoting M2 macrophage polarization.

DSS-induced acute UC was also associated with intestinal dysbiosis. In turn, 7-mix and mix-sup increased bacterial community diversity, restored gut microbial composition, increased the abundance of SCFA-producing bacteria, and increased SCFA concentrations in the feces.

The results showed that the abundance of the seven bacterial strains was dynamically altered over time, and bacterial abundance was inversely correlated with inflammation. However, the seven SCFA-producing bacterial strains colonizing the gut reduced inflammation and improved gut barrier function. Bacterial activity is affected by potential competitive inhibition among strains. Therefore, the ability of commensal bacteria to colonize the gut over time remains difficult to assess.

This study has limitations. The activity of 7-mix and mix-sup was validated using a mouse model of acute UC and cell lines. Therefore, future studies using other murine models of UC are needed, including trinitrobenzene sulfonic acid-induced UC and chronic UC. Moreover, underlying mechanisms should be elucidated.

## Conclusions

A mixture of seven SCFA-producing bacterial strains and the resulting culture supernatant mixture attenuated experimental UC by inducing M2 macrophage polarization and inhibiting JAK/STAT3/FOXO3 axis activation in macrophages. Our approach provides new opportunities to rationally harness live gut probiotic bacteria and metabolites to reduce intestinal inflammation, remodel the gut flora, and expedite the development of safe and efficient treatments for IBD.

### Electronic supplementary material

Below is the link to the electronic supplementary material.

Supplementary Material 1

Supplementary Material 2

## Data Availability

The data supporting the conclusions of this study are available in the NCBI Sequence Read Archive repository under accession numbers PRJNA884653 and PRJNA884670.

## Abbreviations

FMT: fecal microbiota transplantation
IBD: inflammatory bowel disease
SCFA: short-chain fatty acid
UC: ulcerative colitis
CD: Crohn’s disease
IL-10: interleukin-10
LPS: lipopolysaccharide
DSS: dextran sulfate sodium
MPO: myeloperoxidase
ZO-1: zonula occludens 1
JAK: Janus kinase
STAT: signal transducer and activator of transcription
FOXO: forkhead box O
iNOS: inducible nitric oxide synthase
7-mix: seven-strain bacterial mixture
mix-sup: supernatant mixture
hu-FMT: human fecal microbiota transplantation
DAI: disease activity index
CLD: clodronate
HE: hematoxylin and eosin
IHC: immunohistochemistry
PCoA: principal coordinate analysis
ROR-γt: retinoic acid receptor-related orphan receptor gamma t
