# Supplementary material for: Short-chain fatty acid-producing bacterial strains attenuate experimental ulcerative colitis by promoting M2 macrophage polarization via JAK/STAT3/FOXO3 axis inactivation
Source: J Transl Med. 2024 Apr 18;22:369. doi: 10.1186/s12967-024-05122-w (PMC11025230; doi:10.1186/s12967-024-05122-w)
Supplement: Supplementary file 1 — Supplementary Material 1 [file 12967_2024_5122_MOESM1_ESM.docx]

**Supplementary Material**

**Short-chain fatty acid-producing bacterial strains attenuate experimental ulcerative colitis by promoting M2 macrophage polarization via JAK/STAT3/FOXO3 axis inactivation.**

**Materials and methods**

**Sequencing**

Total fecal DNA was extracted using the TIANamp Bacteria DNA Kit and analyzed by NovoGene (Beijing, China). The V3−V4 region of the 16S rRNA gene was amplified by PCR using specific primers (16S V4:515F-806R) with barcodes. The reaction mixture contained 15 µL of Phusion^®^ High-Fidelity PCR Master Mix (New England Biolabs), 2 µM forward and reverse primers, and 10 ng of template DNA in a total reaction volume of 30 µL. Amplification conditions consisted of initial denaturation at 98 °C for 1 min, followed by 30 cycles of denaturation at 98 °C for 10 s, annealing at 50 °C for 30 s, and elongation at 72 °C for 30 s. PCR products were detected by electrophoresis on a 2% agarose gel and purified using the Qiagen Gel Extraction Kit (Qiagen, Germany). Sequencing libraries were generated using the TruSeq^®^ DNA PCR-Free Sample Preparation Kit (Illumina, USA) following the manufacturer’s recommendations, and index codes were added. Library quality was assessed using a Qubit@ 2.0 fluorometer (Thermo Scientific) and the Agilent Bioanalyzer 2100 system. The library was sequenced on the Illumina NovaSeq platform, generating 250 bp paired-end reads. High-quality sequences with ≥ 97% similarity were clustered into operational taxonomic units, as described previously^1^. Species α-diversity was calculated using Chao1 and Shannon indices, and β-diversity was evaluated by principal coordinate analysis (PCoA) using QIIME software. Functional profiles were predicted from 16S rRNA data using Tax4Fun2.

**Strain isolation and culture**

Fecal bacterial isolation and culture were performed as described in our previous studies^2,3^. Briefly, fresh fecal samples were collected from three healthy donors, and each sample was divided into two parts. One part was placed in an anaerobic workstation (LAI-D2, Longyue, Shanghai, China) under anaerobic conditions (90% N_2_, 5% CO_2_, and 5% H_2_), and the other part was grown under aerobic conditions. A fresh sample (1.0 gram) was added to 10 mL of sterile PBS, vortexed, and allowed to stand for 5 min. The fecal suspension was transferred to anaerobic blood culture bottles (BD, BACTECTM Lytic/10 Anaerobic/F Culture Vials, USA) and aerobic blood culture bottles (Autobio, Zhengzhou, China) supplemented with sterile sheep blood and rumen fluid (Elite-Media, Shanghai, China) at 37 °C for 30 days according to the culturomics strategy of Lagier and Yang^4,5^. Then, 1 mL of the bacterial mixture was collected using a sterile syringe and diluted serially from 10 to 10^12^ every 3 days, and the same volume of fresh medium was added to each bottle. One hundred microliters of each dilution were evenly coated on lysogeny broth, brain-heart infusion, or MRS medium using “L” sticks, and a single colony was isolated. Colonies were identified using a matrix-assisted laser desorption/ionization system (Bruker Daltonics, Germany), and the 16S rRNA gene sequences of the strains were determined. The assembled sequences were aligned with the NCBI database (blast.ncbi.nlm.nih.gov). We isolated and cultured three SCFA-producing bacteria: *Streptococcus salivarius* FDAARGOS_771 (GenBank: CP053998.1), *Enterococcus hirae* ATCC 9790 (Taxonomy ID:768486), and *Ligilactobacillus salivarius* DSM 20555 (Taxonomy ID:1423799). *F. prausnitzii* A2-165 (DSM 17677) and *A. muciniphila* (DSM 22959) were purchased from Deutsche Sammlung von Mikroorganismen und Zellkulturen. *Lactobacillus casei* (ATCC 393) and *Clostridium butyricum* (ATCC 19398) were purchased from the American Type Culture Collection (ATCC). These cultures were combined to produce a 7-strain mixture (7-mix).

**Measurement of SCFA concentrations by liquid/gas chromatography‒mass spectrometry (LC-MS/GC-MS)**

Seven samples of bacterial culture supernatants were freeze-dried and resuspended in cold 80% methanol and 0.1% formic acid by vortexing. The samples were incubated on ice for 5 min and centrifuged at 15,000*g* at 4 °C for 20 min. The supernatant containing 53% methanol was diluted with LC-MS grade water, transferred to an Eppendorf tube, and centrifuged at 15,000*g* at 4 °C for 20 min. The supernatant was injected into an LC-MS/MS system (Agilent, USA) to measure SCFA concentrations^6,7^. The raw data files generated by UHPLC-MS/MS were processed using Compound Discoverer version 3.1 (Thermo Fisher). Peaks were matched with the mzCloud (https://www.mzcloud.org/), mzVault, and MassList databases. Statistical analyses were performed using statistical software R version 3.4.3, Python version 2.7.6, and CentOS release 6.6.

A standard curve was constructed using short-chain fatty acid standards. Mouse fecal samples were freeze-dried and resuspended in 0.5% phosphoric acid. The samples were centrifuged at 14,000*g* for 10 min; 800 μL of the supernatant was transferred to a test tube, and an equal volume of ethyl acetate was added. The tube was mixed for 2 min and centrifuged at 14,000*g* for 10 min. Next, 600 μL of the upper organic phase was added to 4-methylpentanoic acid with a final concentration of 500 μM as an internal standard, mixed well, and transferred to the injection port of a GC-MS system (Agilent 7890A/5975C, USA). Fecal SCFA concentrations were measured using the standard curve.

**Supplementary Table 1**. Composition of the LYBHIv4 liquid medium.

| Component | 1 L |
| --- | --- |
| Brain Heart Infusion | 37 g |
| Yeast extract | 5 g |
| D-xylose | 1 g |
| D-fructose | 1 g |
| D-galactose | 1 g |
| Cellobiose | 1 g |
| Maltose | 1 g |
| Sucrose | 1 g |
| L-arabinose | 0.5 g |
| N-acetylglucosamine | 0.5 g |
| L-cysteine | 0.5 g |
| Malic acid | 1 g |
| Sodium sulfate | 2 g |
| Menadione | 20 mg |
| Hemin | 5 mg |
| Tween 80 | 0.5 mL (0.05%) |
| pH 7.2 |  |

**Supplementary Table 2**. Composition of the AKK medium.

| Component | 1 L |
| --- | --- |
| Yeast extract | 10 g |
| Soy peptone | 16 g |
| CaCl_2_·2H_2_O | 0.147 g |
| MgCl_2_·6H_2_O | 0.2 g |
| NH_4_Cl | 0.3 g |
| NaCl | 0.3 g |
| Cysteine | 0.3 g |
| Glucose | 4.5 g |
| Threonine | 0.5 g |
| N-acetyl-glucosamine | 5.5 g |
| Na_2_HPO_4_ | 2.5 g |
| Acidic working solution (1×) | 1 mL |
| Alkaline working solution (1×) | 1 mL |
| Component of acidic working solution (10×) | 100 mL |
| FeCl_2_·4H_2_O | 0.149 g |
| H_3_BO_3_ | 0.0618 g |
| ZnCl_2_ | 0.0682 g |
| CuCl_2_·2H_2_O | 0.017 g |
| MnCl_2_·4H_2_O | 0.099 g |
| CoCl_2_·6H_2_O | 0.119 g |
| NiCl_2_·6H_2_O | 0.0238 g |
| HCl | 0.42 mL |
| Component of alkaline working solution (10×) | 100 mL |
| Na_2_SeO_3_ | 0.0173 g |
| Na_2_WO_4_·2H_2_O | 0.033 g |
| NaMoO_4_·2H_2_O | 0.0242 g |
| NaOH | 0.4 g |

**Supplementary Table 3**. Primer sequences.

| Primer | Forward (5’ to 3’) | Reverse (5’ to 3’) |
| --- | --- | --- |
| m (ZO-1) | CAAAGCCCACCAAGGTCAC | TCTCTTTCCGAGGCATTAGCA |
| m (Occludin) | GTGGAGTTGCGGGAGAGC | TCCCAAGATAAGCGAACCTGCC |
| m (IL-6) | GTTGCCTTCTTGGGACTGATG | ATTGCCATTGCACAACTCTTT |
| m (TNF-α) | TTAGAAAGGGGATTATGGCTCA | ACTCTCCCTTTGCAGAACTCAG |
| m (IL-1β) | AGAGCATCCAGCTTCAAATCTC | CAGTTGTCTAATGGGAACGTCA |
| m (IL-10) | GCTCTTACTGACTGGCATGAG | CGCAGCTCTAGGAGCATGTG |
| m (IL-4) | ATCATCGGCATTTTGAACGAGGTC | ACCTTGGAAGCCCTACAGACGA |
| m (TGF-β) | TGATACGCCTGAGTGGCTGTCT | CACAAGAGCAGTGAGCGCTGAA |
| m (T-bet) | CCACCTGTTGTGGTCCAAGTTC | CCACAAACATCCTGTAATGGCTTG |
| m (IFN-γ) | CAGCAACAGCAAGGCGAAAAAGG | TTTCCGCTTCCTGAGGCTGGAT |
| m (GATA3) | CCTCTGGAGGAGGAACGCTAAT | GTTTCGGGTCTGGATGCCTTCT |
| m (IL-4) | ATCATCGGCATTTTGAACGAGGTC | ACCTTGGAAGCCCTACAGACGA |
| m (IL-5) | GATGAGGCTTCCTGTCCCTACT | TGACAGGTTTTGGAATAGCATTTCC |
| m (RORγt) | GTGGAGTTTGCCAAGCGGCTTT | CCTGCACATTCTGACTAGGACG |
| m (IL-17A) | CAGACTACCTCAACCGTTCCAC | TCCAGCTTTCCCTCCGCATTGA |
| m (FOXP3) | CCTGGTTGTGAGAAGGTCTTCG | TGCTCCAGAGACTGCACCACTT |
| m (iNOS) | GAGACAGGGAAGTCTGAAGCAC | CCAGCAGTAGTTGCTCCTCTTC |
| m (IL-12) | ACGAGAGTTGCCTGGCTACTAG | CCTCATAGATGCTACCAAGGCAC |
| m (IL-23) | CATGCTAGCCTGGAACGCACAT | ACTGGCTGTTGTCCTTGAGTCC |
| m (CD206) | GTTCACCTGGAGTGATGGTTCTC | AGGACATGCCAGGGTCACCTTT |
| m (JAK1) | CTGTCTACTCCATGAGCCAGCT | CCTCATCCTTGTAGTCCAGCAG |
| m (JAK2) | GCTACCAGATGGAAACTGTGCG | GCCTCTGTAATGTTGGTGAGATC |
| m (STAT3) | AGGAGTCTAACAACGGCAGCCT | GTGGTACACCTCAGTCTCGAAG |
| m (FOXO3) | CCTACTTCAAGGATAAGGGCGAC | GCCTTCATTCTGAACGCGCATG |
| h (Occludin) | ATGGCAAAGTGAATGACAAGCGG | CTGTAACGAGGCTGCCTGAAGT |
| h (ZO-1) | GTCCAGAATCTCGGAAAAGTGCC | CTTTCAGCGCACCATACCAACC |
| h (JAK2) | CCAGATGGAAACTGTTCGCTCAG | GAGGTTGGTACATCAGAAACACC |
| h (STAT3) | CTTTGAGACCGAGGTGTATCACC | GGTCAGCATGTTGTACCACAGG |
| h (FOXO3) | TCTACGAGTGGATGGTGCGTTG | CTCTTGCCAGTTCCCTCATTCTG |
| h (TNF-a) | CTCTTCTGCCTGCTGCACTTTG | ATGGGCTACAGGCTTGTCACTC |
| h (IL-6) | AGACAGCCACTCACCTCTTCAG | TTCTGCCAGTGCCTCTTTGCTG |
| h (IL-1β) | CCACAGACCTTCCAGGAGAATG | GTGCAGTTCAGTGATCGTACAGG |
| Universal eubacteria | GGTGAATACGTTCCCGG | TACGGCTACCTTGTTACGACTT |
| *F. prausnitzii* | GGAGGAAGAAGGTCTTCGG | AATTCCGCCTACCTCTGCACT |
| *C. butyricum* | ATGGGTTAGGCAAGCAGAAA | GCCTTCCACTGTAGGGTAATATG |
| 1. *muciniphila* | GCGTAGGCTGTTTCGTAAGTCGTGTGTGAAAG | GAGTGTTCCCGATATCTACGCATTTCA |
| *S. salivarius* | CACGCCATGCTGGAAGTG | GCGATGAGCCAAGCTGAAG |
| *L. salivarius* | CGAAACTTTCTTACACCGAATGC | GTCCATTGTGGAAGATTCCC |
| *E. hirae* | CTTTCTGATATGGATGCTGTC | TAAATTCTTCCTTAAATGTTG |
| *L. casei* | AGCAGTAGGGAATCTTCCA | ATTYCACCGCTACACATG |

**Supplementary Table 4**. Correlation analysis between bacterial abundance and inflammatory markers. We used data from the Inflammatory Bowel Disease Multi’omics Database. Data represent correlation matrices and *p*-values.

| Bacterial abundance | Modified Baron’s score | C-reactive protein | Erythrocyte sedimentation rate |
| --- | --- | --- | --- |
| p_Proteobacteria | 0.62, 0.005** | -0.15, 0.53 | 0.82, ﹤0.001*** |
| f_Enterobacteriaceae | 0.65, 0.003** | -0.06, 0.77 | 0.91, ﹤0.001*** |
| s_*Dialister_pneumosintes* | 0.34, 0.16 | -0.04, 0.85 | 0.74, 0.0002*** |
| s_*Clostridium_symbiosum*_ATCC_14940 | -0.23, 0.36 | 0.98, ﹤0.001*** | -0.15, 0.52 |
| g_*Eisenbergiella* | -0.26, 0.29 | 0.97, ﹤0.001*** | -0.23, 0.31 |
| s_*Bacteroides_caccae* | -0.31, 0.21 | 0.93, ﹤0.001*** | -0.24, 0.30 |
| s_*Parabacteroides_distasonis* | -0.08, 0.75 | 0.92, ﹤0.001*** | -0.25, 0.29 |
| g_*Tyzzerella* | 0.08, 0.76 | 0.81, ﹤0.001*** | -0.21, 0.36 |
| s_*Holdemania_filiformis* | -0.04, 0.87 | 0.80, ﹤0.001*** | -0.11, 0.64 |
| s_*Bacteroides_eggerthii* | 0.50, 0.036* | -0.06, 0.78 | 0.34, 0.14 |
| g_*Escherichia-Shigella* | 0.49, 0.04* | -0.11, 0.64 | 0.16, 0.48 |
| s_*Shigella_sonnei* | 0.49, 0.04* | -0.11, 0.64 | 0.17, 0.48 |
| s_*Prevotella*_sp_109 | 0.50, 0.03* | -0.13, 0.57 | -0.15, 0.50 |
| p_Firmicutes | -0.26, 0.30 | 0.09, 0.70 | -0.53, 0.015* |
| f_Lachnospiraceae | -0.27, 0.27 | 0.37, 0.10 | -0.52, 0.017* |
| g_*Faecalibacterium* | -0.17, 0,51 | -0.51, 0.02* | 0.12, 0.59 |
| f_Akkermansiaceae | -0.11, 0.66 | -0.10, 0.67 | -0.14, 0.56 |
| f_Ruminococcaceae | -0.11, 0.66 | -0.31, 0.18 | -0.02, 0.96 |
| f_Bifidobacteriaceae | -0.25, 0.31 | -0.22, 0.35 | -0.18, 0.43 |
| g_*Roseburia* | 0.01, 0.49 | -0.30, 0.21 | -0.18, 0.62 |

**Supplementary Table 5**. Relative quantification (log peak area) of short-chain fatty acids in the culture supernatant of seven bacterial strains. Values are means ± standard deviations.

| Strain | Acetate | Propionate | Butyrate | Isobutyrate |
| --- | --- | --- | --- | --- |
| *F. prausnitzii** | 6.92 ± 0.05* | 5.74 ± 0.06* | 7.47 ± 0.04* | 6.78 ± 0.08* |
| *A. muciniphila^$^* | 7.58 ± 0.37 | 6.31 ± 0.11**^$^* | 7.54 ± 0.02*^$^* | 6.22 ± 0.12* |
| *S. salivarius^#^* | 7.20 ± 0.03*^#^ | 6.45 ± 0.07**^$#^* | 7.31 ± 0.06**^$#^* | 6.06 ± 0.16* |
| *L. casei^@^* | 8.40 ± 0.05*^#@^ | 6.52 ± 0.09**^$^* | 8.48 ± 0.29**^$#@^* | 6.02 ± 0.07* |
| *C. butyricum^%^* | 7.84 ± 0.11*^#@^ | 6.56 ± 0.08**^$#^* | 7.76 ± 0.03**^$#%^* | 6.07 ± 0.18* |
| *L. salivarius^&^* | 8.07 ± 0.05*^#@^ | 6.57 ± 0.06**^$#^* | 8.15 ± 0.12**^$#%&^* | 6.14 ± 0.12* |
| *E. hirae* | 7.98 ± 0.09*^#@^ | 6.53 ± 0.08**^$^* | 7.65 ± 0.20^@&^ | 6.07 ± 0.07* |

Note: Each strain has a unique symbol indicating a significant difference (*p*<0.05) in the concentration of short-chain fatty acids between this and other strains.

**Supplementary Figures**

**
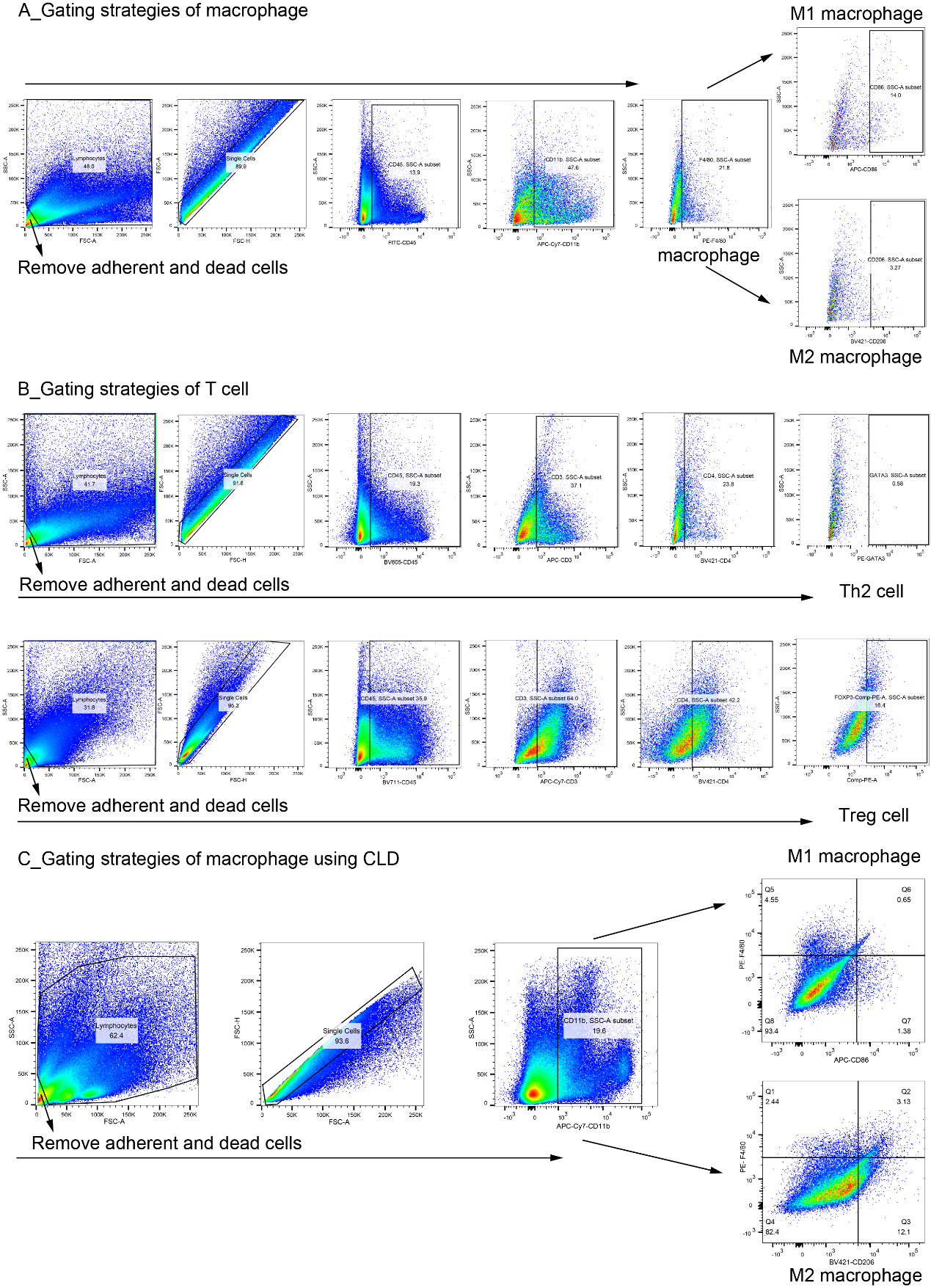
**

**Supplementary Figure 1.** Gating strategies for macrophages (**A**, **C**) and T cells (**B**).


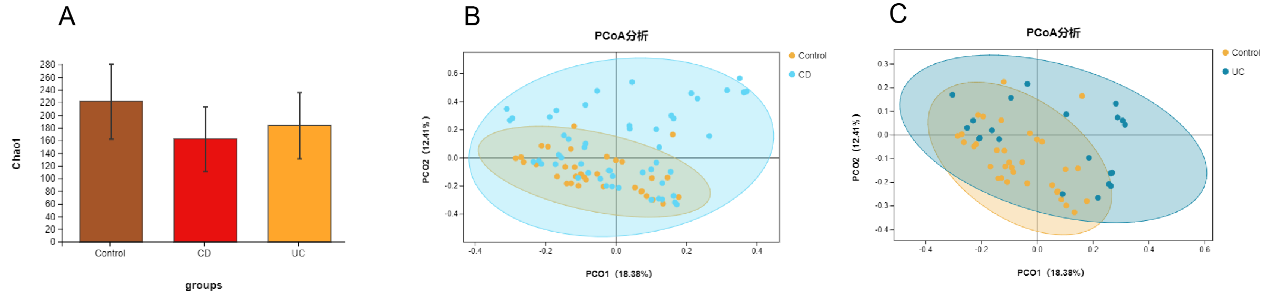


**Supplementary Figure 2.** α-diversity (Chao1 index) (**A**) and β-diversity (principal coordinate analysis) (**B**, **C**) in the feces of patients with Crohn’s disease (CD) or ulcerative colitis (UC) compared with healthy subjects. We used the data from the Inflammatory Bowel Disease Multi’omics Database.


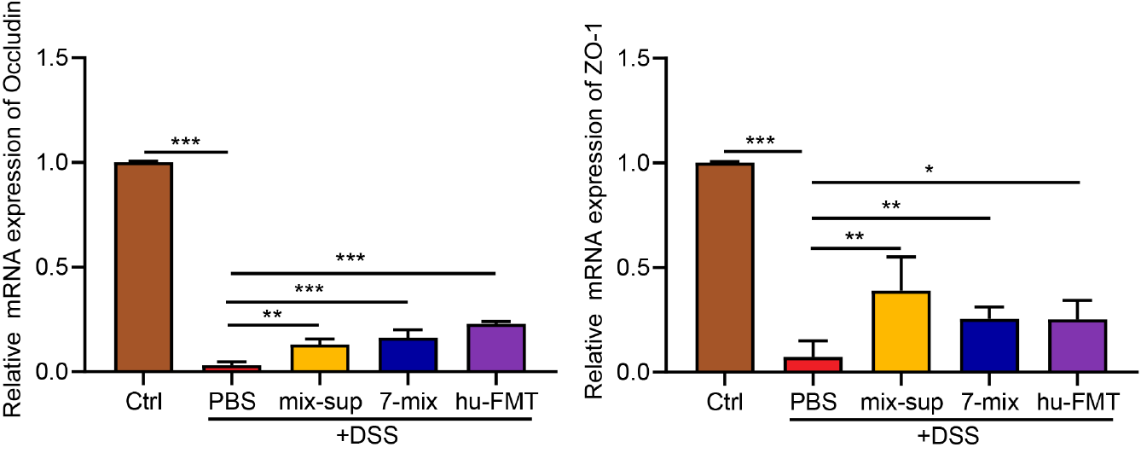


**Supplementary Figure 3.** qRT-PCR analysis of the relative mRNA expression of occludin and ZO-1 in colitic mice treated with PBS, bacterial mixture (7-mix), culture supernatant mixture (mix-sup), or human fecal microbiota transplantation (hu-FMT) relative to control mice.

**
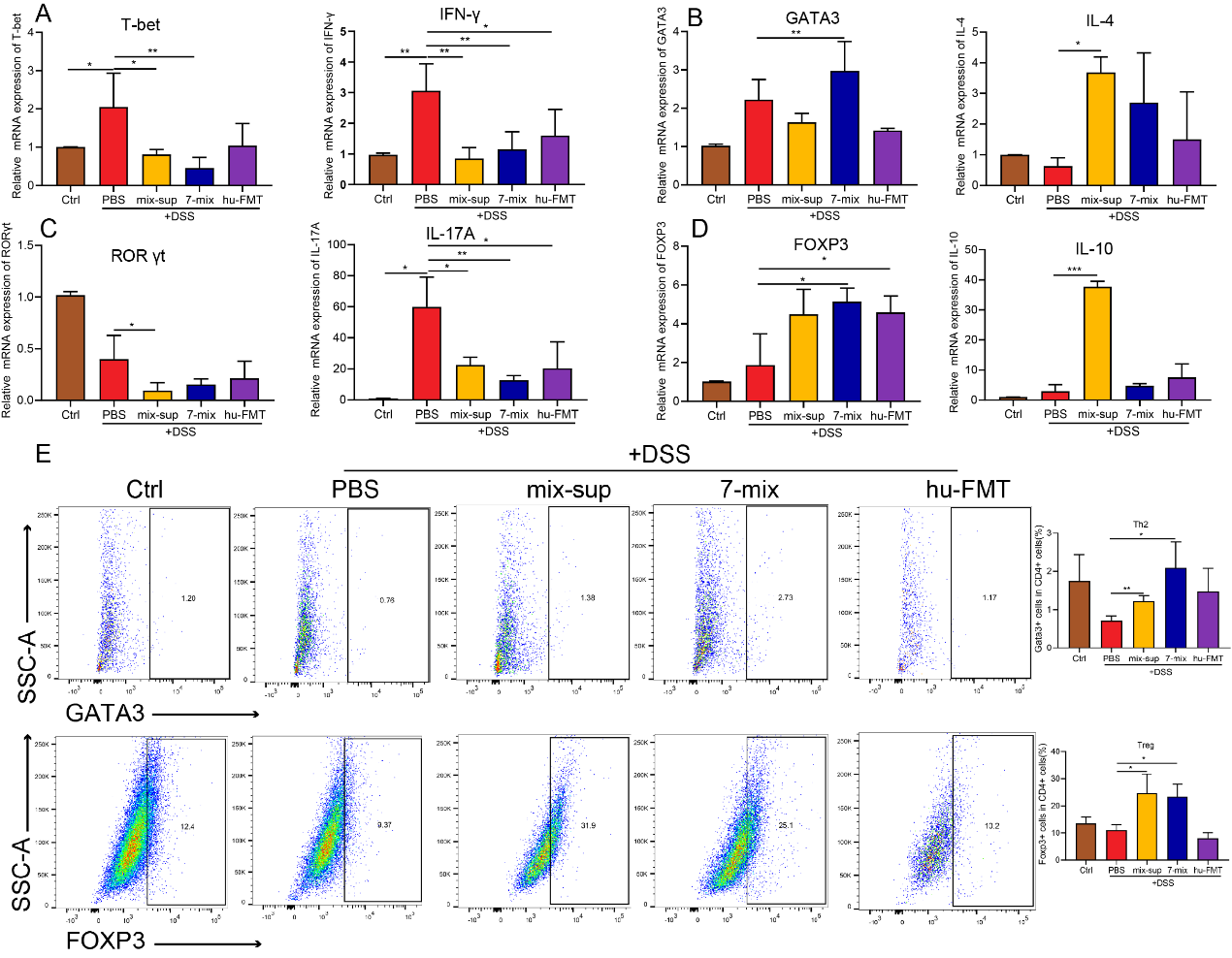
**

**Supplementary Figure 4.** Relative mRNA expression of Th1-associated cytokines T-bet and IFN-γ (**A**), Th2-associated cytokines GATA3 and IL-4 (**B**), Th17-associated cytokines RORγt and IL-17A (**C**), and Treg-associated cytokines FOXP3 and IL-10 (**D**) in the colon of colitic mice relative to control mice. **E**. Flow cytometry analysis of the number of GATA3^+^ Th2 and Foxp3^+^ T regulatory cells in colonic lamina propria (**E**). Data are means ± standard deviations, N = 3-4. **P* < 0.05, ***P* < 0.01, ****P* < 0.001.

**
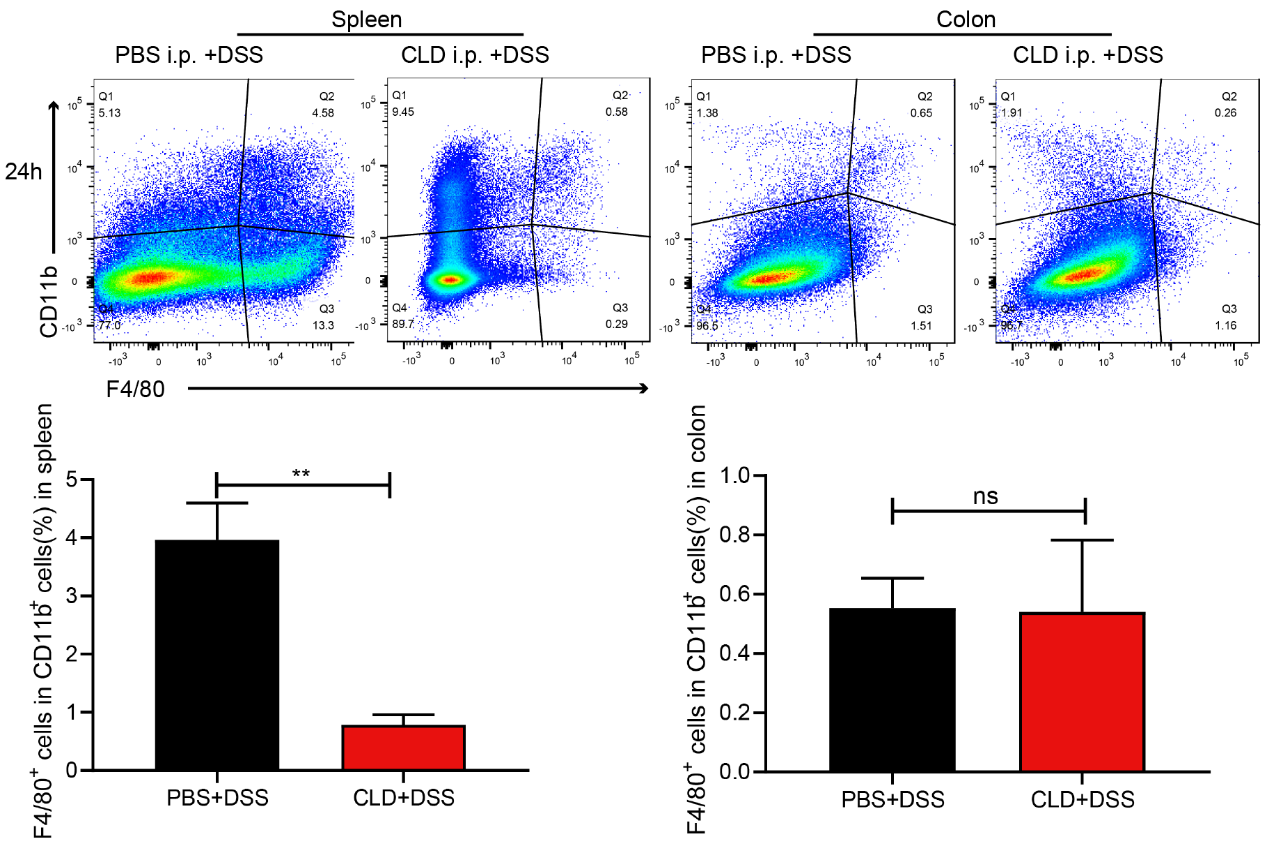
**

**Supplementary Figure 5.** Number of F4/80^+^ macrophages in the spleen and colon of colitic mice treated or not with clodronate (CLD) liposomes.

**
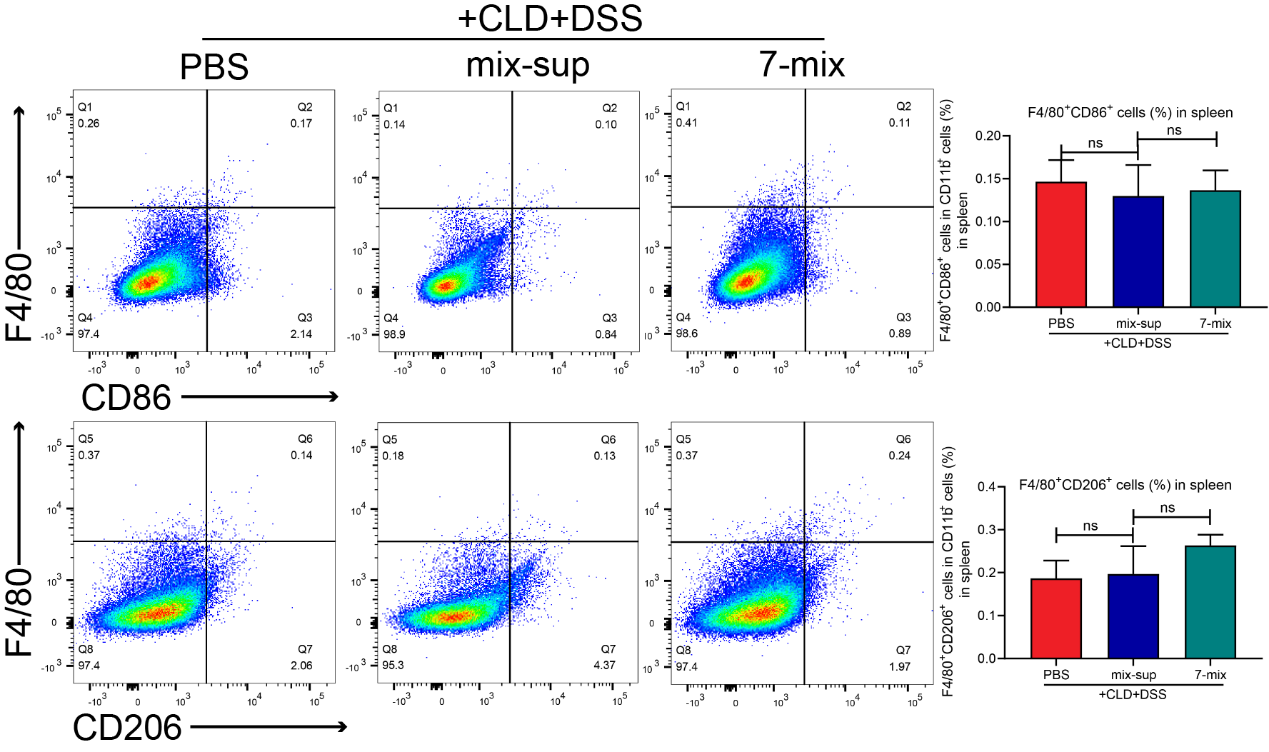
**

**Supplementary Figure 6.** Number of F4/80^+^ macrophages in the spleen of CLD-treated colitic mice who received PBS, a bacterial mixture (7-mix), or culture supernatant mixture (mix-sup).

**References**

1. Edgar RC. UPARSE: highly accurate OTU sequences from microbial amplicon reads. *Nature methods* 2013; **10**(10): 996-8.

2. Zhao H, Peng Y, Cai X, et al. Genome insights of Enterococcus raffinosus CX012922, isolated from the feces of a Crohn's disease patient. *Gut pathogens* 2021; **13**(1): 71.

3. Zhao H, Wang J, Peng Y, et al. Genomic insights from Paraclostridium bifermentans HD0315_2: General features and pathogenic potential. *Front Microbiol* 2022; **13**: 928153.

4. Lagier J-C, Dubourg G, Million M, et al. Culturing the human microbiota and culturomics. *Nat Rev Microbiol* 2018; **16**: 540-50.

5. Chang Y, Hou F, Pan Z, et al. Optimization of Culturomics Strategy in Human Fecal Samples. *Front Microbiol* 2019; **10**: 2891.

6. Barri T, Dragsted LO. UPLC-ESI-QTOF/MS and multivariate data analysis for blood plasma and serum metabolomics: effect of experimental artefacts and anticoagulant. *Analytica chimica acta* 2013; **768**: 118-28.

7. Want EJ, O'Maille G, Smith CA, et al. Solvent-dependent metabolite distribution, clustering, and protein extraction for serum profiling with mass spectrometry. *Analytical chemistry* 2006; **78**(3): 743-52.
