# Supplementary material for: Short-chain fatty acid-producing bacterial strains attenuate experimental ulcerative colitis by promoting M2 macrophage polarization via JAK/STAT3/FOXO3 axis inactivation
Source: J Transl Med. 2024 Apr 18;22:369. doi: 10.1186/s12967-024-05122-w (PMC11025230; doi:10.1186/s12967-024-05122-w)
Supplement: Supplementary file 2 — Supplementary Material 2 [file 12967_2024_5122_MOESM2_ESM.docx]

Dear Editor-in-Chief,

I hope this letter finds you well. I am writing in response to the valuable feedback provided by the reviewers for our recently submitted manuscript titled “Short-chain fatty acid-producing bacterial strains attenuate experimental ulcerative colitis by promoting M2 macrophage polarization via JAK/STAT3/FOXO3 axis inactivation” [JTRM-D-23-10610]. I appreciate the time and effort the reviewers invested in evaluating our work, and I am grateful for their insightful comments. Accordingly, we have carefully revised the manuscript. All amendments are highlighted in yellow in the revised manuscript. In addition, point-by-point responses to the comments are on the subsequent pages.

I believe that these revisions have substantially improved the manuscript, aligning it more closely with the standards of *Journal of Translational Internal Medicine*. I am confident that the modifications address the concerns raised by the reviewers and contribute to the overall scholarly value of the paper.

I would like to express my gratitude for the opportunity to revise and resubmit our manuscript. I am confident that the revisions made will meet the expectations of both the reviewers and the editorial team. I look forward to the possibility of having our work reconsidered for publication in *Journal of Translational Medicine*. The content was carefully revised by a native English-speaking editor specialized in editing manuscripts written by non-native English-speaking scientists.

Thank you for your time and consideration.

Sincerely,

Yuqiang Nie

**Point-to point responses**

**Replies to Reviewer**

Reviewer #1: The manuscript “Short-chain fatty acid-producing bacterial strains attenuate experimental ulcerative colitis by promoting M2 macrophage polarization via JAK/STAT3/FOXO3 axis inactivation” submitted by Zhao et al, discusses the short-chain fatty acid-producing bacterial strains in the setting of UC. The authors also use the mouse model to a sort of validate the studies. Even though the authors have used several different methods to evaluate their hypothesis, the results are not well written and their findings are not represented well enough for publication.

**Replies to Reviewer #1**: Thank you for your feedback and for taking the time to review our manuscript. We appreciate your comments regarding the clarity of our results and the representation of our findings. We have employed rigorous methodologies to evaluate our hypothesis, including multiple assays and the use of the mouse model to validate our findings. We are committed to revising our manuscript to address any deficiencies in clarity and representation of our findings.

Reviewer #2, Venkat Sundar Gadepalli: The article is well-written with clear hypothesis and the experimental design to assess the specific steps to reach a conclusion.

**Replies to Reviewer #2**: We sincerely appreciate your acknowledgment of the clarity of our hypothesis and the robustness of our experimental design. Your recognition of our experimental approach is encouraging to us. We aimed to ensure that each step of our study was meticulously designed to support our conclusions effectively. We are grateful for your time and effort in reviewing our work, and we are pleased to hear that you found our article well-written.

**Specific Comments**

1. Does Mouse model of acute ulcerative colitis (UC) have similar 16s RNA profiles to those patient database analysis performed on https://ibdmdb.org database?

Response: Yes, the 16S rRNA profiles were similar in mouse model and patients. The results are summarized as follows: an increase in the relative abundance of g_*Bacteroides*, g_*Parabacteroides*, p_Proteobacteria, and g_*Escherichia* in the UC group; however, an increase in the relative abundance of g_*Lactobacillus*, g_*Lachnospiraceae*, g_*Faecalibacterium* and g_*Akkermansia* in the treatment groups.

2. It is noted that 16sRNA seq analysis was performed using qime and tax4fun. The github repository provided in the tax4fun article seems to be inactive, have they considered an alternative for researchers to reproduce the analysis results when data is made available on NCBI?

Response: This repository in the Tax4fun has been archived by the owner on Aug 10, 2022. Assessing the functionality and redundancy of microbiota is a major challenge in microbiology. Tax4Fun2 that can quickly predict the functional spectrum and functional redundancy of prokaryotes based on 16S rRNA gene sequences. Thank you very much for pointing out here. We have corrected the sentence in the revised manuscript.
